# Alleviation of ADHD symptoms by non-invasive right prefrontal stimulation is correlated with EEG activity

**DOI:** 10.1016/j.nicl.2020.102206

**Published:** 2020-02-06

**Authors:** Uri Alyagon, Hamutal Shahar, Aviad Hadar, Noam Barnea-Ygael, Avi Lazarovits, Hadar Shalev, Abraham Zangen

**Affiliations:** a Department of Life Sciences and the Zlotowski Centre for Neuroscience, Ben-Gurion University of the Negev, Beer-Sheva, Israel; b Psychiatry Department, Soroka Medical Center, Beer-Sheva, Israel

**Keywords:** Repetitive transcranial magnetic stimulation, Attention deficit hyperactivity disorder, Electroencephalogram, Biomarker, Clinical-trial

## Abstract

Attention deficit hyperactivity disorder (ADHD) is a prevalent disorder with effective pharmacological treatment that benefits most patients. However, about one-third fail to benefit while others search non-pharmacological alternatives, and for those options are scarce. One alternative treatment option is to alter abnormal right prefrontal cortex (rPFC) activity, given that rPFC abnormality has been repeatedly implicated in ADHD neurophathology. Here, we evaluated whether targeting the rPFC with multiple sessions of repetitive transcranial magnetic stimulation (rTMS), which can modulate neuronal excitability, activity, and plasticity in a non-invasive manner, will affect clinical symptoms in adults suffering from ADHD. Concomitantly, we used EEG to characterize electrophysiological alterations induced by treatment and to search for correlation between baseline neuronal activity and clinical response.

Forty-three drug free adults with ADHD were randomized to receive either Real, Active Control, or Sham treatment (13 females, age ranging 21-46; *n* = 15, 14, 14, respectively), and underwent three weeks of daily high-frequency (18 Hz) stimulation sessions. We found that Real treatment was safe and resulted in significant improvement of symptoms (η^2^_p_ = 0.34; Cohen's d_(against Sham)_ = 0.96; Cohen's d_(against AC)_ = 0.68; *p* = 0.00085). Furthermore, based on EEG recorded within the first treatment session we established a novel biomarker, composed of the Alpha and Low-gamma power, which highly correlated the magnitude of the clinical outcome (*r* = 0.92, *p* = 0.0001).

Taken together, the results of this pilot study indicate safety and effectiveness of rTMS directed to the rPFC for treatment of adult ADHD patients. The biomarker is suggested to reflect the responsiveness of the cortex to this rTMS intervention. Following validation of the results in larger samples, this study may represent a step towards a non-pharmacological treatment for adults with ADHD using EEG-based selection of optimal candidates for treatment.

## 1 Introduction

Attention Deficit Hyperactivity Disorder (ADHD) is characterized by poor attention, impulsivity, hyperactivity and emotional-motivational dysregulation (Sonuga-Barke, 2005), affecting 7.2% of children and 3.4% of adults worldwide (Fayyad et al., 2007; Thomas et al., 2015). Taking together the fact that almost 30% of participants find current pharmacological treatments ineffective or intolerable (Biederman et al., 2004), and the lack of treatment producing long-term effects, alternative medical options are needed. One such alternative is non-invasive brain stimulation using transcranial magnetic stimulation (TMS), which may induce long-term alleviation of symptoms by targeting the underline neuropathology.

TMS enables to modulate cortical excitability, to focally alter brain activity, and to promote plasticity at the network level (Fitzgerald et al., 2006; Pascual-Leone et al., 2005). Multiple sessions of repetitive TMS (rTMS) protocols are investigated as potential treatments for various conditions, and are gradually becoming a viable clinical neuromodulation intervention (Lefaucheur et al., 2014). For example, rTMS directed to the left prefrontal cortex (PFC) has been cleared by the FDA for the treatment of medication-resistant depression (Levkovitz et al., 2015; O'Reardon et al., 2007) and rTMS directed to the medial prefrontal and cingulate cortices was recently cleared by the FDA for the treatment of obsessive compulsive disorder (Carmi et al., 2019).

ADHD is associated with deficits in key domains of executive functions, especially in response inhibition, maintenance of sustained attention, working memory and planning (Willcutt et al., 2005). In accordance, it is characterized by multiple functional and structural neural network abnormalities, most prominently of frontal networks (Rubia et al., 2014). For example, meta-analyses of whole-brain voxel-based morphometry (VBM) or fMRI studies during inhibitory control and attentional tasks, found that the right ventrolateral and dorsolateral prefrontal cortices (VLPFC and DLPFC) are part of fronto-basal-ganglia under-functioning networks in ADHD (Hart et al., 2013; Norman et al., 2016), making the right PFC a potential target for rTMS treatment (but see also Samea et al., 2019).

Non-invasive brain stimulation was administered previously in ADHD, but primarily in studies including children and adolescents (Bandeira et al., 2016; Breitling et al., 2016; Cao et al., 2018, 2019; Gómez et al., 2014; Nejati et al., 2017; Soff et al., 2017; Soltaninejad et al., 2019; Sotnikova et al., 2017), case reports (Niederhofer, 2008, 2012), single session protocols (Bloch et al., 2010; Cosmo et al., 2015; Jacoby and Lavidor, 2018) or stimulation protocol of 3-5 sessions (Allenby et al., 2018; Cachoeira et al., 2017). A meta-analysis of transcranial direct current stimulation (tDCS) in ADHD (Salehinejad et al., 2019) found that bi-lateral or left DLPFC (but not right VLPFC) tDCS and anodal (but not cathodal) tDCS significantly improved inhibitory control. On the other hand, and in accordance with the above, a review of neurostimulation in ADHD [20] found that most TMS studies agreed that increasing the excitability of the right DLPFC through high frequency rTMS or decreasing the excitability of the left DLPFC through low frequency rTMS can improve ADHD symptoms, but with mixed results. One cross-over rTMS study (Bloch et al., 2010) found that a single session improved attentional index in ADHD patients. Another study found that 6 weeks of rDLPFC high frequency rTMS combined with atomoxetine is more effective than rTMS alone or atomoxetine alone (Cao et al., 2018). Yet, other sham-controlled studies failed to find significant differences between the groups, possibly due to focality of stimulation missing potentially effective targets or simultaneous stimulation of opposing targets (Paz et al., 2017; Weaver et al., 2012). As such, we attempted to target the rPFC unilaterally with a TMS coil that produces a wide distribution of the magnetic field, and that affects both the right VLPFC and the right DLPFC.

It is important to note that stimulation protocols, especially those that use high-frequency stimulation, involve a degree of physical discomfort that may induce bias to the treatment outcome. This is especially relevant during sham-controlled studies, and thus the use of active stimulation to control for the influence of TMS-related sensation upon sham effect is advocated by the guidelines for TMS usage and research (Lefaucheur et al., 2014). In addition, treatment protocols burden the patient heavily in terms of time and money, and not all patients are expected to benefit. Thus, a method that will allow assessment of individual suitability for treatment, and potential clinical gains per individual, is much in need (for examples see Arns et al., 2008; Tenke et al., 2011; Dinteren et al., 2015; Sun et al., 2016; Silberstein et al., 2017). Preferably, such a method will enable accurate prediction of the clinical outcome as early and with as little disturbance to the patient as possible.

**Fig. 1:**
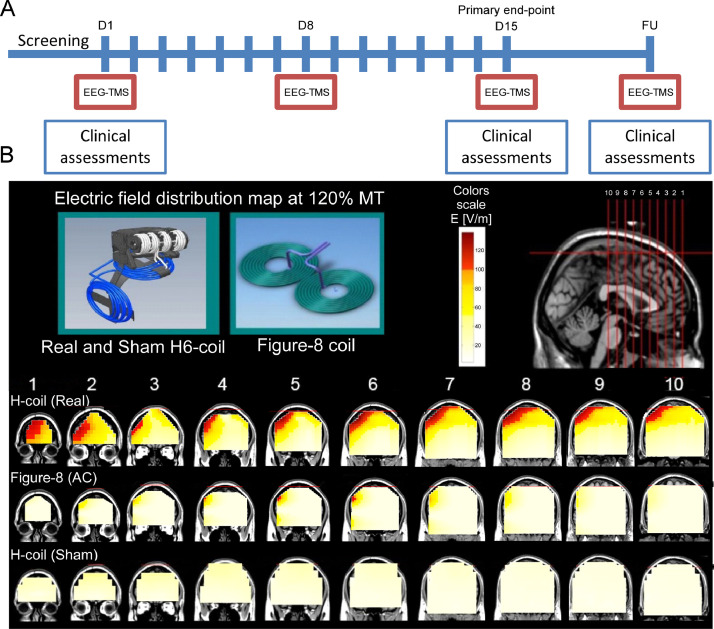
Study design and rTMS coils. (A) Participants received 5 daily sessions per week for 3 weeks (15 sessions total), and a maintenance treatment session during the follow-up visit 1 month later. Three sessions (D1, D15, and FU) included clinical assessments, and 4 included EEG recordings (D1, D8, D15, and FU). (B) The colored maps, overlaid on MRI images, describe the absolute magnitude of the electrical field induced by the TMS coils at intensity equivalent to 120% of the resting motor threshold, as measured in a phantom model of the human head (Roth et al., 2007). Red pixels indicate regions with field intensity above the threshold for neuronal activation, which was set to 100 V/m. Coils were positioned 5 cm anterior and 2 cm lateral to the typical motor hotspot. Top right panel shows the brain slices (1 cm between each slice) used to present the field maps. D – Day; FU – Follow Up; AC – Active Control. (For interpretation of the references to color in this figure legend, the reader is referred to the web version of this article.)

To account for these considerations, we designed a semi-blinded (see *study design* for clarification), randomized study that investigate the clinical, behavioral and electrophysiological influences of high frequency rTMS treatment directed to a wide portion of the rPFC, including the DLPFC and VLPFC (Fig. 1A). We compared the results to those of a group receiving sham stimulation and a group receiving active control (AC) stimulation. For all groups we used similar temporal pattern of stimulation and similar number of pulses, but the Sham coil induced a parallel to scalp, non-penetrating magnetic field, while the AC coil induced a focal supra-threshold field directed to the midway between the DLPFC and the VLPFC (Fig. 1B). Note that the term "active control" is used in accordance with brain stimulation trials (Lefaucheur et al., 2014) rather than clinical equivalence trials (Jones et al., 1996). In addition, we attempted to identify electroencephalography (EEG) based markers which are altered by the rTMS treatment or ones that are correlated with the clinical outcome. We investigated brain activity recorded in 3 conditions with increasing level of disturbance to the participant: resting state, in response to single magnetic pulses (TMS evoked potential; TEP), and during the rTMS treatment session.

## 2 Materials and methods

### 2.1 Participants

Fifty-two TMS-naive participants suffering from ADHD (mostly students; 13 females), age ranging 21-46, were recruited over 3 years using ads or mass university email. Participants received information concerning the study requirements over the telephone and were further screened by a senior psychiatrist using a semi structured interview (SCID) based on DSM-V criteria to verify ADHD diagnosis and to rule out psychiatric comorbidities. No minimum score of the Conners’ Adult ADHD Rating Scale (CAARS) or other questionnaire was required. Participants suffering from any additional axis I or II diagnosis including anxiety, depression (major or bi-polar), obsessive compulsive disorder, personality disorder (including anti-social), or substance abuse; taking anti-psychotic, anti-depressive or mood stabilizers; have own or first-degree relative's epileptic history; suffered from significant neurological disorder or insult in the past, or those who could not tolerate rTMS stimulation, were excluded from the study. Participants were required to refrain from taking any psychostimulant medication for a week before, and during the rTMS treatment phase. All participants provided written informed consent and did not receive financial compensation. The experimental protocol was approved by the ethics committee of the Soroka University Medical Center and registered at the NIH (ClinicalTrials.gov:NCT01737476).

### 2.2 Study design

Participants were randomly assigned to the Real, AC, or Sham group. The Real and Sham coils were built together into a single helmet. Sham treatment was designed to mimic the auditory artifacts evoked by the active coil, without stimulating the brain itself (Levkovitz et al., 2015). Randomization of the respective operation mode of the stimulation (Real or Sham) was determined by a pre-programmed magnetic treatment card individually assigned to each subject through the double-blind randomization process. AC stimulation was administered using a separated coil (see below), and as all participants were TMS naive, they were told that stimulation may be either real or sham. Thus, participants were completely blinded to group assignment, while TMS operators were blinded to the Real/Sham assignment only (hence the term "semi-blinded"). Participants received 15 rTMS treatment sessions over 3 weeks, and a maintenance treatment session during a follow-up (FU) visit 1 month after the last treatment session. Prior to and following each treatment session, participants completed a short computerized assignment (Stern et al., 2016) designed to activate the relevant brain pathways. This was done in accordance with previous studies suggesting that engagement of the relevant circuitry may increase clinical response to rTMS (Beaulieu and Milot, 2018; Carmi et al., 2019, 2017; Dinur-Klein et al., 2014; Isserles et al., 2013; Lieshout et al., 2017) . Note that delivering this assignment during the treatment is not possible due to distraction caused by the stimulation. Clinical assessments were conducted 3 times: pre-treatment, post-treatment, and FU.

### 2.3 TMS devices and procedure

TMS was delivered using a Magstim Rapid^2^ stimulator (Magstim, UK) inducing biphasic pulses. The Real stimulation was delivered using an H6-coil which was specially designed, based on the principles of the H-coil family, to unilaterally stimulate wide portions of the right PFC including the VLPFC and the DLPFC (Roth et al., 2002; Roth and Zangen, 2014; Zangen et al., 2005). The Sham coil was encased in the same helmet with the Real coil (as described above) and induced auditory artifact but a non-penetrating electromagnetic field (Levkovitz et al., 2015). The AC treatment was delivered using a Figure-8 coil (Magstim, UK) with 70mm wings; handle oriented approximately 45° from the midline. Participants were required to use earplugs during TMS sessions. Individual left hand RMT was measured at the beginning of each treatment (Levkovitz et al., 2015) and the coil was then moved 5 cm anteriorly and 2 cm laterally from the motor hot spot to target the right PFC (All coils where moved in a similar manner). These placement parameters were set according to the H6-coil design and, importantly, when applied with figure-8 coil, do not target directly the DLPFC (5-6 cm anterior to the motor hot spot; between electrode F4 and AF4) (Fitzgerald et al., 2009) or the VLPFC (5 cm anterior and 4 cm lateral to the motor hot spot; electrode F8) (Mottaghy et al., 2002; Vanneste and Ridder, 2012), which are the two most implicated pre-frontal targets in ADHD related network. Thus, AC stimulation was expected to produce a focal off-target stimulation compared to the Real stimulation, with resembling acoustic and somatosensory sensations. Stimulation included 40, 2 s long, 20 s apart, 18 Hz TMS trains (total of 1440 pulses per session) at stimulator power output of 120% of Resting Motor Threshold (RMT).

### 2.4 Clinical assessment

During every clinical assessment participants completed the CAARS (self-report, long version) (Conners et al., 1999a; Erhardt et al., 1999), Barkely Adult ADHD Rating Scale (BAARS-IV) (Barkley, 2010), Behavioral Rating Inventory for Executive Functioning (BRIEF-A) (Roth et al., 2005), and Beck Depression Inventory (BDI) (Beck et al., 1961). Primary outcome measure was defined as the change in ADHD total symptoms according to the CAARS norms from pre- to post-treatment (D1 to D15; CAARS scores are standardized according to age and gender; normal distribution, mean = 50, SD = 10) (Conners et al., 1999b). Secondary outcome measures were response rate, defined as 25% or higher reduction in total ADHD symptoms score of the CAARS questionnaire (Cheng et al., 2007; Durell et al., 2013; Amiri et al., 2012; Newcorn et al., 2009; Montoya et al., 2014), and other subscales of the CAARS, BAARS-IV, BRIEF-A, and BDI.

### 2.5 Behavioral tasks

We used Mindstreams – a validated cognitive battery (Dwolatzky et al., 2003; Schweiger et al., 2003) to assess treatment related behavioral changes across an array of ADHD related cognitive domains and tasks, including: attention, executive function, information processing speed, memory, and the Stroop task. The Mindstreams battery delivers a composite score for Stroop performance which adjusts for speed-accuracy tradeoffs (Osman et al., 2000), and is computed as accuracy divided by response time of the incongruent condition. This score, though only partially measuring the Stroop effect (congruent-incongruent), was previously found to distinguish between ADHD participants and controls (Schweiger et al., 2007). Mindstreams was administered three times, coupled with the clinical assessment (D1, D15, FU). Mindstream's scores are standardized according to age- and education-specific normative data (Doniger, 2008) (normal distribution, mean = 100, SD = 15).

Additionally, we used the Stop Signal Task (see detailed methods in supplementary materials) to assess acute (single session) and prolonged treatment effects on behavioral inhibition. This task was delivered eight times (before and after the treatments in D1, D8, D15, and FU).

### 2.6 TEP protocol

TEP was delivered using the figure-8 coil to all participants, independently of the treatment coil identity. Fifty single TMS pulses, at stimulator power output of 120% of RMT, with an inter-pulse interval of 5 s, were delivered to the treatment area before and after treatments in D1, D8, D15 and FU.

### 2.7 EEG recordings and processing

Recording and preprocessing methods concerning segmentation, filtration, and removal of TMS related and non-related artifacts are detailed in the supplementary methods. Briefly:

EEG was acquired in D1, D8, D15 and FU using a TMS-compatible 64-channel amplifier (ANT Ltd.) during resting state, TEP procedure, and treatment (in this order; eyes closed during all conditions). Preprocessing was conducted using EEGlab (Delorme and Makeig, 2004). Resting state data was transformed to the frequency domain and analyzed as power in specific bands, while TEP activity was segmented around the TMS pulse and analyzed in the time domain according to conventional TEP components (Premoli et al., 2014; Rogasch et al., 2014).

Treatment data was extracted from the time periods between the trains (Inter-train intervals, ITI) and segmented into 2 s epochs starting 1 s after train's ending to avoid TMS related artifacts induced by the stimulation and ceasing 5 s before the upcoming train to avoid activity alterations caused by anticipation to the next train (total of 7 segments per ITI). The data was then transformed to the frequency domain.

The in-treatment based biomarker was computed as Alpha (8-14.5 Hz) to Low-gamma (30–40 Hz) activity power ratio (see Results) based on the first post-train ITI segments recorded in the first treatment session (seconds 1–3 after each train's ending). This timeframe was chosen to depict the acute influence of the stimulation on the EEG while avoiding TMS related artifacts.

To eliminate the possibility that prefrontal activity may originate from posterior sources (Hagemann et al., 2001), we computed spectral density using two reference schemes: average reference (AVR) and Current Source Density (CSD) (Hagemann, 2004). Results using AVR are presented in the main text, while results using CSD are presented in supplementary Fig. 3. Both methods lead to similar outcomes and conclusions.

### 2.8 Statistical analysis

Sample size was set to 54 (45 before 20% estimated dropout rate) assuming a medium-large effect size (Levkovitz et al., 2015) and requiring power of 0.95 at a significance level of 5% in a 3 by 2 model of mixed ANOVA. ANOVAs were conducted using STATISTICA software (version 13; TIBCO Soft Inc.). All statistical inference was performed using two tailed tests requiring a-priory alpha level of 5%.

Clinical and behavioral measures were analyzed using 2 way mixed model ANOVAs with time (pre, post treatment) as within subjects factor and group (Real, AC, Sham) as between subjects factor. Post hoc significance tests were Bonferroni corrected. Response rates were tested using Fisher exact non parametric test. Correlations between the clinical primary outcome measure and the behavioral scores in the Mindstreams cognitive battery were computed using Pearson linear coefficient. Significance values were Bonferroni corrected for 18 tests (6 cognitive scores × 3 groups).

All electrophysiological data were tested, unless detailed otherwise, using non-parametric permutation analysis (Monte-Carlo method) implemented in FieldTrip (Oostenveld et al., 2011). Multiple tests due to electrodes number were corrected using cluster based permutation test.

The biomarker correlative value was tested using a whole scalp permutation analysis with *r* statistic between the marker's power in each electrode and the primary outcome measure of total ADHD symptoms of the CAARS.

The inter-hemispheric balance analysis investigated if the observed changes in ADHD symptoms are the resultant of an asymmetric phenomenon, reflecting the activity balance between the two hemispheres, and not the absolute power in each individual hemisphere. It was computed based on partial correlations between the marker's power in each electrode and the CAARS total ADHD symptoms, controlling for the marker's power in the contra-lateral symmetric electrode. By that, we eliminated (partialled out or residualized (Wheeler et al., 1993)) the influence of common activity shared by the two hemispheres. Permutation tests are not suited for such a multi-step process, thus the partial correlations where tested using parametric tests and controlled using False Detection Rate (FDR) method for 54 electrodes, excluding the midline channels (Benjamini and Hochberg, 1995). Following results of the first analysis, additional inter-hemispheric balance model was conducted for the Alpha activity from the resting state EEG of the first treatment session, targeting the 8 pre-frontal most implicated channels (FC4, FC2, F4, F2 and the paired left channels; FDR corrected).

Further details concerning statistical methods for testing of secondary behavioral and electrophysiological outcome measures (Mindstreams, investigation of marker's components, and treatment related effects on TEP and resting state activity) can be found in supplementary methods.

## 3 Results

Fifty-two subjects were enrolled to the study following screening by a psychiatrist, and assigned to the Real, AC or the Sham group (*n* = 20, 16, 16, respectively). Forty-three subjects completed the treatment phase and were included in the final analysis (*n* = 15, 14, 14, respectively; see supplementary Fig. 1 and supplementary Table 1 for a consort diagram and detailed sample size for each analysis). No baseline differences were found between the groups in demographic data, or primary and secondary measures (Table 1).

**Table 1:** Baseline demographic clinical and behavioral characteristics.

	Sham	AC	Real	p value
Female\Male	11\3	10\4	13\2	0.6
Age	27.64 (1.58)	26.13 (0.59)	26.62 (0.66)	0.58
CAARS ADHD total symptoms (*t* score)	78.14 (3.27)	79.20 (2.00)	73.62 (3.85)	0.41
BAARS total ADHD score	45.5 (3.76)	47.00 (1.93)	45.54 (2.59)	0.91
BRIEF-A GEC	65.00 (2.34)	67.14 (3.05)	69.4 (2.01)	0.47
BDI	8.36 (2.16)	4.00 (0.88)	6.66 (0.71)	0.12

Means and standard errors are detailed. CAARS - Conners' Adult ADHD Rating Scale; BAARS - Barkely Adult ADHD Rating Scale-IV; BRIEF-A - Behavioral Rating Inventory for Executive Functioning; GEC - Global Executive Composite scale; AC – Active Control.

### 3.1 Clinical and behavioral effects

**Fig. 2:**
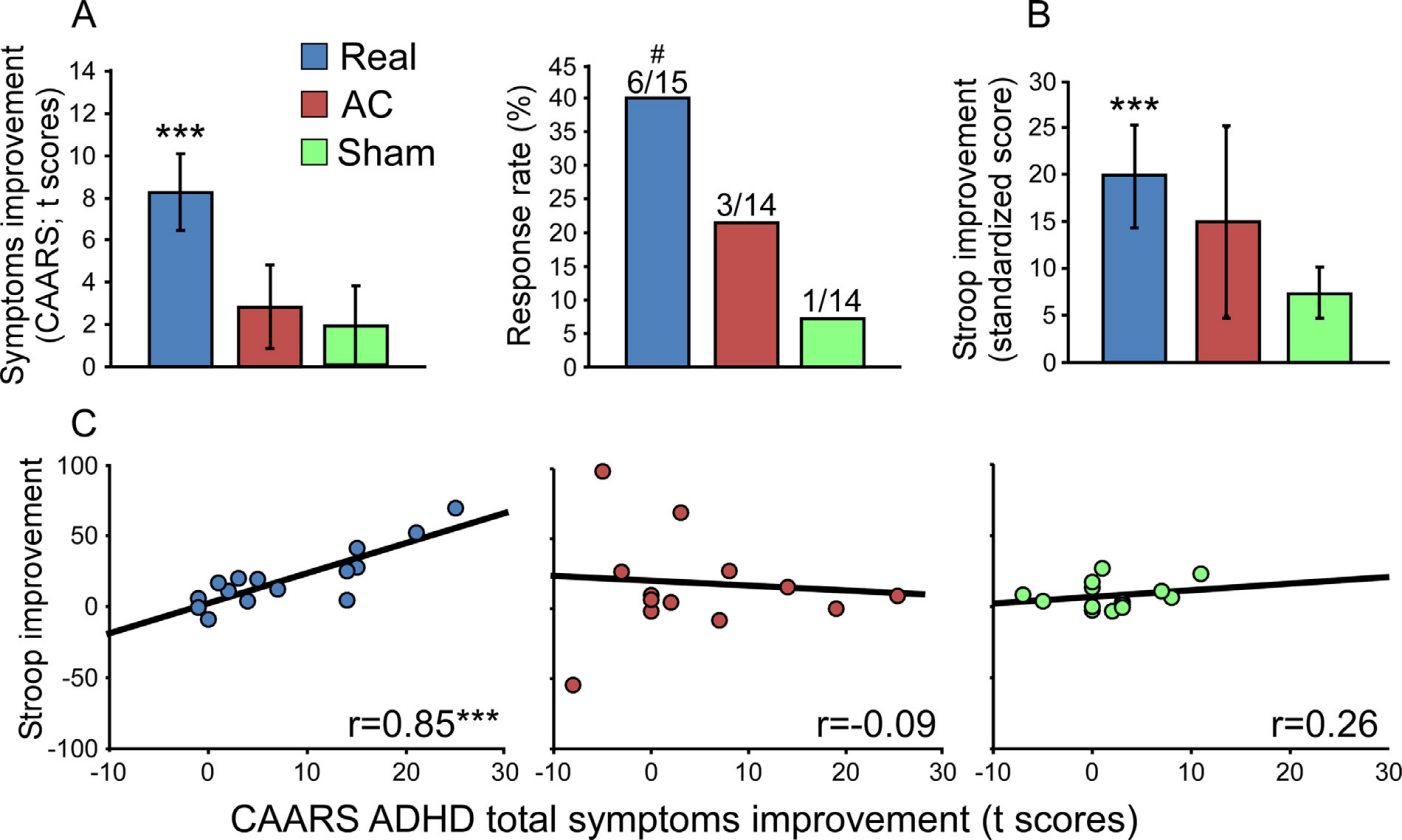
Clinical and behavioral results. (A) Symptom improvement and response rates after 3 weeks of treatment. (B) Stroop performances improvement after 3 weeks of treatment, and with correlation to symptoms improvement (C). ***p_c_ < 0.001 between pre- and post-treatment results of the Real group; ^#^*p* = 0.08 compared to the Sham group. CAARS - Conners' Adult ADHD Rating Scale; AC – Active Control.

One subject from the AC group experienced a seizure during the 3^rd^ treatment session and terminated participation (see supplementary case report). No additional adverse events were reported other than transient headaches and scalp discomfort localized to the stimulation area. ANOVA of the primary outcome measure (Fig. 2A) revealed significant main effect of Time (*F*_(1,39)_ = 15.60, *p* = 0.0005; η^2^_p(partial)_ = 0.29), along with a significant Time X Group interaction (*F*_(2,39)_ = 3.45, *p* = 0.042; η^2^_p_ = 0.15). The mean improvement scores (and SE) in ADHD total symptoms were 8.27 ± 1.83, 2.84 ± 1.96, 1.86 ± 1.90 for the Real, AC and Sham groups, respectively. Post-hoc comparisons revealed that only the Real group showed a significant improvement (*F*_(1,39)_ = 20.45, *p*_c(corrected)_ = 0.00085; η^2^_p_ = 0.34; Cohen's d_(against Sham)_ = 0.96; Cohen's d_(against AC)_ = 0.68). In addition, analysis of the secondary outcome measure of response rate revealed a marginally significant differences between the Real and Sham groups (40.0% vs 7.1% improvement; *p* = 0.08), but not between Real and AC groups (40.0% vs 21.4% improvement; *p* = 0.43; Fig. 2A). Analysis of FU scores revealed a similar, but non-significant patterns of Time X Group interaction effect (*F*_(2,32)_ = 1.14, n.s; 9.55 ± 3.17, 3.09 ± 3.17, 4.61 ± 2.92 of mean improvement in ADHD total symptoms for the Real, AC and Sham groups, respectively), and response rates (36%, 25%, 15%, respectively). Importantly, rPFC stimulation did not induce alteration in BDI in this ADHD population (*F*_(2,39)_ = 1.17, n.s; supplementary Fig. 2D), and improvement in ADHD symptoms was not mediated by BDI change (see supplemental analysis).

Analysis of the various CAARS subscales and the BAARS-IV revealed greater improvement in the Real group across domains, albeit only the hyperactivity/impulsivity domain demonstrated significant differences compared to the control groups (supplementary Fig. 2A and B). In the behavioral measures of the Mindstreams computerized battery (supplementary Fig. 2C), similar trends for greater improvement in the Real group were observed, especially in the Stroop task (Fig. 2B). ANOVA of the change in Stroop performances revealed a significant effect for Time (*F*_(1,26)_ = 18.39, *p* = 0.0002; η^2^_p_ = 0.41), along with a marginally significant Time X Group interaction (*F*_(1,26)_ = 3.95, *p* = 0.057; η^2^_p_ = 0.13). Post-hoc comparisons found improvement in Stroop performances in the Real group relative to the Sham group (*F*_(1,26)_ = 21.21, p_c_ = 0.00057; η^2^_p_ = 0.45), but not relative to the AC group (*F*_(1,26)_ = 0.19, n.s). Moreover, across the Mindstreams domains, the Real group demonstrated high correlation with clinical improvement (supplementary Fig. 2C, insertion), which again was most pronounced in the Stroop task (Fig. 2C). More specifically, the improvement of Stroop performances and the reduction of ADHD symptoms significantly correlated within the Real group (*r*_(13)_ = 0.85, *p_c_* = 0.001), but not within the AC (*r*_(11)_ = −0.09, n.s) or the Sham (*r*_(11)_ = 0.26, n.s) groups. Conversely, analysis of stopping times in the Stop Signal Task did not reveal differences between the groups, as a ubiquitous improvement in task performance (presumably training effect due to multiple task repetitions) in all groups was observed (supplementary Fig. 2E). Finally, results from the BRIEF-A questionnaire indicated improvement in distinct executive functions, albeit none of those reached significance (supplementary Fig. 2F).

### 3.2 Biomarkers correlated with treatment outcome

**Fig. 3:**
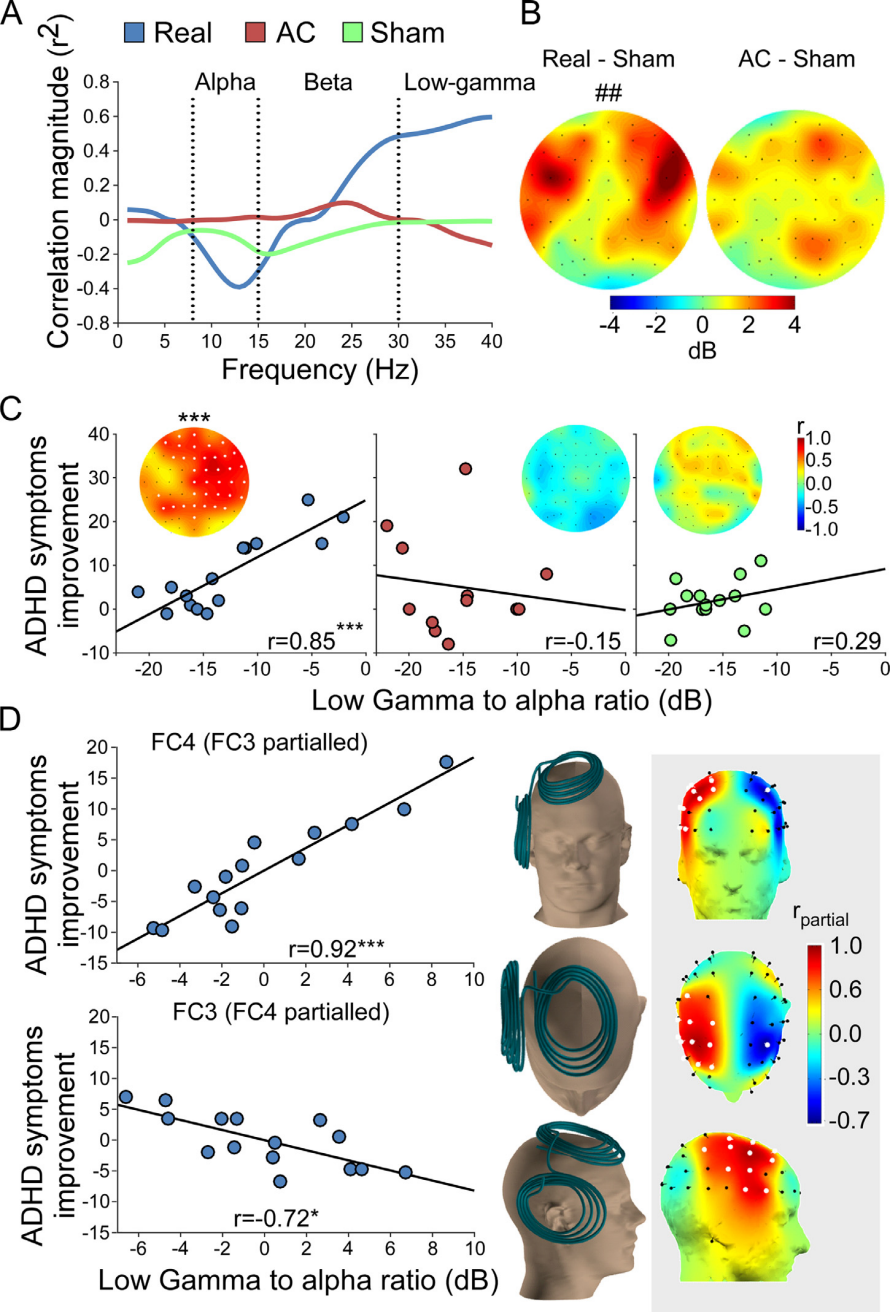
Treatment based biomarker. (A) Linear correlations as a function of Group and Frequency, expressed as explained variance (*r*^2^; correlation sign is maintained), between activity power measured at channel FC4 during treatment (under the stimulation area) and symptoms improvement. (B) Topographic plots of the averaged group differences in the power of the marker (Low-gamma to Alpha power ratio), as a contrast between the active groups and the Sham group. (C) Topographic plots and scatter plots (at channel FC4) of the linear correlations between the marker's power and improvement of ADHD symptoms. (D) Scatter plots (for channels FC4 and FC3) and head plots of the inter-hemispheric balance model in the Real group. Electrodes for which correlations are significant are colored white. ^##^*p_uc_* < 0.01 at channel FC6; *p_c_ < 0.05; ***p_c_ < 0.005. AC – Active Control. (For interpretation of the references to color in this figure legend, the reader is referred to the web version of this article.)

In an attempt to identify the electrophysiological correlates of the stimulation we investigated EEG activity during the inter-train interval of the treatment to seek for activity alternations caused by the rTMS train (Allen et al., 2007; Pasley et al., 2009), in addition to the more traditional single TMS pulse approach (Rogasch and Fitzgerald, 2013; Sun et al., 2016). We did not reveal clear correlations between clinical outcomes and treatment related alternations following 3 weeks (as detailed below and in supplementary materials). However, we did identify two activity components observed under the stimulation area during the inter-train intervals of the first TMS session of the Real (but not of the control groups) which were correlated with the clinical outcome. Alpha activity was found to be negatively correlated (*r*_(n=15)_ = −0.56, *p_c_* = 0.035), while Low-gamma positively correlated (r_(n=15)_ = 0.74, *p_c_* = 0.012) with the improvement of symptoms (Fig. 3A and supplementary Fig. 3A). These activity components were used to form an EEG marker calculated as the power ratio between the two frequency bands. Distinguished spatial patterns of elevated marker activity were found in the two active TMS groups when compared with Sham activity (Fig. 3B, supplementary Fig. 3B, and also supplementary Fig. 4A for better 3D visualization). While AC subjects demonstrated a roundly shaped and relatively narrow locus of enhanced marker activity (*p*_uc(uncorrected)_ = 0.12 at channel AF4), the Real group subjects' activity was more widespread but accentuated at its maximal point (*p*_uc_ = 0.007 at channel FC6).

**Fig. 4:**
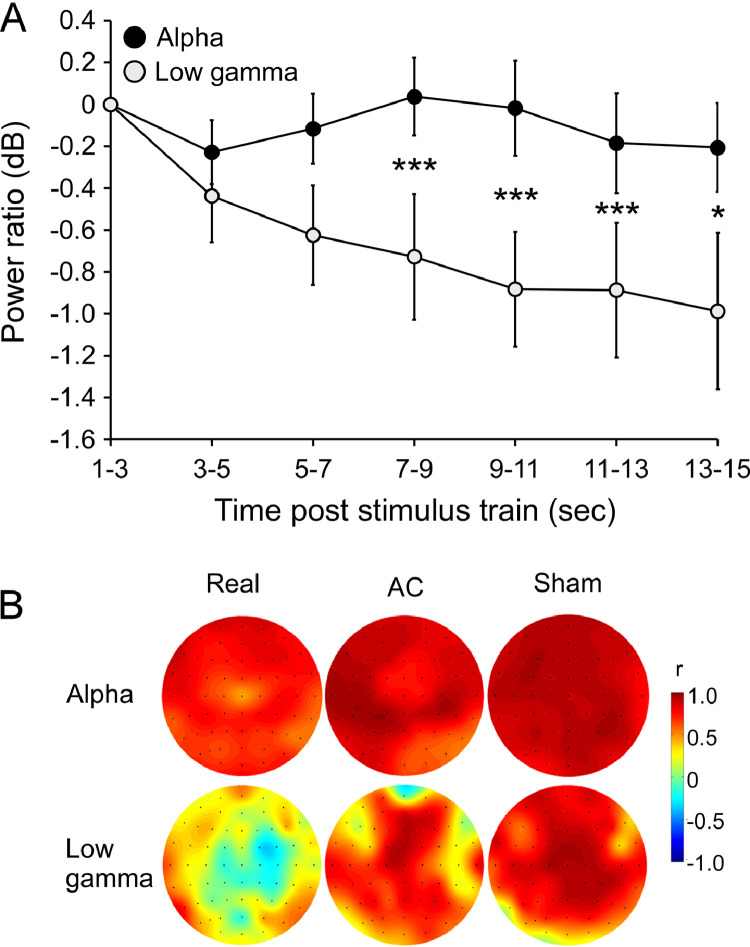
Characterization of the marker's components. (A) Activity dynamics during the ITI for the Alpha and Low-gamma components observed under stimulation area (channel FC4) as a function of time after the stimulus train in the Real group. Power in all time points is expressed as a ratio relative to the first post-train (1-3 s) segment. (B) Correlations between Alpha and Low-gamma power during ITI and resting state in the different groups. *p_c_ < 0.05, ***p_c_ < 0.001. AC – Active Control. (For interpretation of the references to color in this figure legend, the reader is referred to the web version of this article.)

Moreover, a whole scalp correlation analysis between the marker and ADHD total symptoms improvement revealed a significant broad cluster of positive linear correlations in the Real group alone. This finding was most prominent in channels under the stimulation area (Fig. 3C, supplementary Fig. 3C), and observed using either AVR (cluster of 38 channels, *r*_FC4(_*_n_*_=15)_ = 0.85, *p_c_* = 0.003) or CSD (cluster of 33 channels, *r*_FC4(_*_n_*_=15)_ = 0.85, *p_c_* = 0.002). Along with the locus of positive linear correlation seen in the right hemisphere under stimulation area, a moderated correlation pattern was also observed in the left hemisphere (supplementary Fig. 4B). This may be a result of an asymmetric phenomenon like the abnormal brain asymmetry characterizing ADHD (Hale et al., 2009, 2010; Keune et al., 2011, 2015), masked by common activity shared by the two hemispheres (Wheeler et al., 1993). We thus conducted an inter-hemispheric balance analysis to uncover the direct correlation between the marker's power and ADHD symptoms in each electrode (see statistical analysis). The analysis revealed a negative partial correlation between the marker's power and ADHD symptoms improvement in the left frontal area paralleling the stimulation site, both for AVR (*r*_FC4(_*_n_*_=15)_ = 0.92, *p_c_* = 0.0001; *r*_FC3(_*_n_*_=15)_ = −0.72, *p_c_* = 0.02; Fig. 4D) and CSD (*r*_FC4(_*_n_*_=15)_ = 0.91, *p_c_* = 0.0002; *r*_FC3(_*_n_*_=15)_ = −0.64, *p_c_* = 0.049; supplementary Fig. 3D) analyses. The inter-hemispheric balance analysis improved correlation power in additional 12.4% (72.2% and 84.6% of explained variance for the single and dual channel models, respectively). Importantly, remarkable similarity seems to exist between the spatial distribution of correlation magnitude in the model and the stimulation area of the H6 coil over the scalp placement (Fig. 3D).

Next, in order to characterize the nature of the marker's components, we tested their dynamics in response to the stimulation trains, and in comparison to resting state activity. ANOVA of brain activity dynamics within the ITI revealed a significant Time X Frequency interaction (*F*_(5,90)_ = 2.62, *p* = 0.03; η^2^_p_ = 0.13). The slopes of the averaged activity power show that while Low-gamma activity decays gradually after the train, Alpha activity stays relatively stable (Fig. 4A). Post hoc analysis revealed significant differences in power reduction between the frequency bands, starting 7 s after the train. Correlations between activity during the first treatment (first post-train segments of the inter-train intervals) and resting state (just prior to the first treatment and before any stimulation had been delivered) showed a robust pattern of high positive Alpha correlations over the whole scalp in all treatment groups. This pattern was observed also in the Low-gamma band activity of the Sham group, but attenuated in the AC group and abolished in the Real group, especially in electrodes under the stimulation area (Fig. 4B).

**Fig. 5:**
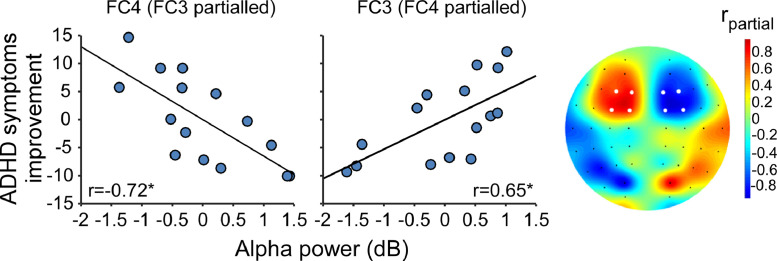
Resting state-based biomarker. Inter-hemispheric balance analysis of partial correlations between Alpha power during resting state before the first treatment and improvement of ADHD symptoms in the Real group. Scatter plots are shown (left) for electrodes FC3 and FC4 as well as topographic plot of the whole head. Electrodes for which correlations are significant are colored white. *p_c_ < 0.05. (For interpretation of the references to color in this figure legend, the reader is referred to the web version of this article.)

As Alpha activity between treatment and resting state was highly correlated, we conducted another inter-hemispheric balance model using the EEG Alpha activity of the Real group during resting state. This analysis revealed a prefrontal locus of significant correlation between activity power at resting state before the first treatment and change in ADHD total symptoms after 3 weeks of treatment (Fig. 5). These partial correlations were negative in the right hemisphere (AVR: *r*_FC4(_*_n_*_=15)_ = −0.72, *p_c_* = 0.0128; CSD: *r*_FC4(_*_n_*_=15)_ = −0.66, *p_c_* = 0.025) and positive in the left (AVR: *r*_FC3(_*_n_*_=15)_ = 0.65, *p_c_* = 0.013; CSD: *r*_FC3(_*_n_*_=15)_ = 0.47, p_c_ = 0.15).

### 3.3 Electrophysiological alterations induced by 3 weeks of rTMS treatment

**Fig. 6:**
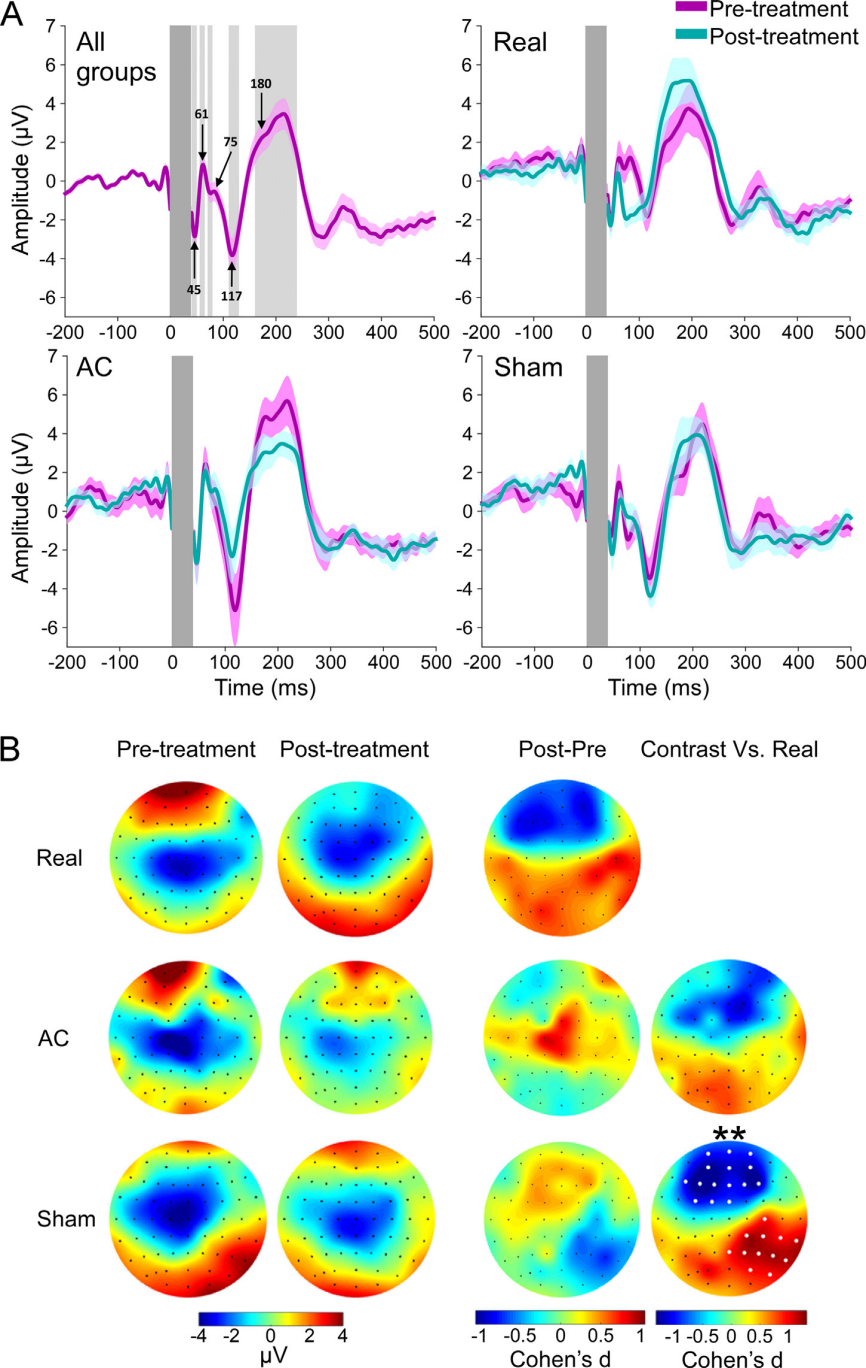
Influence of rTMS treatment on TEP. (A) Averaged baseline TEP from all participants (upper left panel; channel F4) demonstrated typical components of N45, P60, N75, N100, and P180. Data for each individual group, Pre (D1) and Post (D15) treatment, is also presented (upper right and lower panels). Deleted time window of artifact is marked by dark gray, TOIs are marked by light gray, and shaded area around the TEP curves marks the SEM. (B) Topographic plots of the averaged TEPs at the N75 TOI are presented for Pre- and Post-treatment, for Post- minus Pre-treatment, and for the contrast between the control groups and the Real group (expressed as effect size; Cohen's d). Electrodes for which differences are significant are colored white, **p_c_ < 0.01. AC – Active Control. (For interpretation of the references to color in this figure legend, the reader is referred to the web version of this article.)

In order to examine the effect of stimulation on frontal excitability, we compared pre- and post-treatment TEP's. In agreement with former publications (Premoli et al., 2014; Rogasch et al., 2014), we identified a TEP curve observed under the stimulation area (channel F4), with a typical shape of N45, P60, N75, N100 and the P180 components (Fig. 6A). Permutation analysis of Time X Group interaction in these components’ time windows of interest (TOIs) revealed significant frontal cluster in the N75 TOI (*p* = 0.039, channels: AF3-4, AFz, F-4, Fz, FC3, FCz; not shown). Further, decomposition of the N75 effect to its sources revealed a simple Time by Group contrast between the Real and Sham groups containing 2 significant clusters: one frontal (15 channels, *p* = 0.0072) and the other right parietal-occipital (12 channels, *p* = 0.023) (Fig. 6B). The frontal cluster demonstrated treatment induced reduction in the N75 amplitude in the Real compared to the control groups, and had clear ipsi-lateral and contra-lateral loci to the stimulation area. No significant clusters were spotted in the Real/AC contrast. In the P180 TOI we observed a local Time X Group interaction in two electrodes placed under the stimulation area (channel F4, *p_uc_* = 0.028; channel AF4, *p_uc_* = 0.049), but no significant cluster was identified (supplementary Fig. 5). In addition, no further clusters were identified in the other TOIs.

Analysis of the resting state activity before and after 3 weeks of treatment revealed a significant effect on power in the beta band seen in a cluster of electrodes not under the stimulation area in the AC but not in the Real group. This seems to be unrelated to the clinical effect itself, hence results are detailed in the supplementary materials (supplementary Fig. 6).

## 4 Discussion

Overall, the use of rTMS over the rPFC was safe and effective. A single incident of seizure was observed in the AC group, but no additional serious side effects were reported. We found that 3 weeks of daily Real rPFC stimulation can induce alleviation of adults’ ADHD symptoms, compared to AC and Sham stimulation. The treatment effect size (Cohen's d_(against Sham)_ = 0.96; Cohen's d_(against AC)_ = 0.68), measured as symptoms improvement, was comparable to that reported in sham-controlled randomized trials of ADHD pharmacotherapies (Cheng et al., 2007; Faraone et al., 2004; Faraone and Biederman, 2002; Schachter et al., 2001), but was diminished after 1 month of follow-up, and the response rates (40%, 7.1%, 21.4% for the Real, Sham, and AC groups) were relatively modest. Taken together, the clinical results indicate that rTMS directed to the rPFC may serve as an alternative treatment to those adults suffering from ADHD and do not benefit, or cannot tolerate the side effects of existing pharmacological treatments (Biederman et al., 2004) . Further investigation is needed to clarify if the clinical outcome can be promoted by extending the treatment period (which was shorter compared to that of MDD and OCD) (Carmi et al., 2019; Levkovitz et al., 2015), and if maintenance treatments are needed to preserve the clinical effect (Benadhira et al., 2017; Richieri et al., 2013).

The alleviation of ADHD symptoms in the Real group was accompanied by and correlated with a modest improvement in the Stroop composite score, which was previously found to be reduced in adult ADHD patients (Schweiger et al., 2007). The correlation found here between clinical improvement (subjectively reported by the participants) and improvement in performance (objectively measured using a computerized task) supports the validity of the findings. Additionally, taking into account that ADHD participants are reported to exhibit hypo-activation of the right VLPFC during performance of the Stroop and other inhibitory tasks (Hart et al., 2013) suggests that treatment improvement may be mediated by VLPFC modulation induced by the multiple treatment sessions. Nevertheless, given that the Stroop was the only task influenced by the treatment, and the unconventional manner of the Stroop composite score (computed based on the accuracy and reaction time of the incongruent condition alone), it is hard to conclude which cognitive ability was altered by the treatment.

Our study included a two-fold control system, Sham and AC stimulation, to eliminate potential confounds and placebo effects (Lefaucheur et al., 2014). Here, AC was used to mimic the physical sensation of the Real stimulation, including muscular spasms proximate to the target area, which are absent from the Sham stimulation. Given that both Sham and AC groups did not show significant improvement in symptoms, it is less-likely that the clinical effects produced in the Real group resulted from a non-specific muscular, auditory, visual (apparatus shape), or any other confounding factor associated with the stimulation. Nevertheless, as no control is perfect, the greater peripheral stimulation produced by the wide H6-coil compared to focal Figure-8 coil may have augmented the therapeutic effect. Additionally, it should be noted that response rates highlighted therapeutic response in the Real group when compared with the Sham group, but not with the AC group. This is likely due to the impoverished statistical power of the tests being used. However, taking into account variation between subjects and the lack of neuronavigation in this study, it is possible that in few subjects AC stimulation affected relevant brain areas and led to alleviation of symptoms.

Additionally we found a pattern of the rPFC TEP that closely resembles previous TMS-EEG data (Premoli et al., 2014; Rogasch et al., 2014), but did not correlated with the clinical outcome. More specifically, analysis of the evoked neural signals from the designated TEP protocol revealed significant reduction in the N75 amplitude following 3 weeks of treatment in the Real group when compared with the Sham group. These findings of an enduring treatment-induced neural phenomena reflect neuroplastic effect of multiple TMS treatment sessions, and is likely to have intervened in the excitatory-inhibitory interplay of the stimulated neural tissue (Noda et al., 2017). Interestingly, alternations in the global TEP response (global field mean potential), occurring in the same time range of the N75 (60–90 ms after the magnetic pulse) were recently reported in Alzheimer patients following 2 weeks of high frequency rTMS treatment (Koch et al., 2018). However, to the best of our knowledge, no functional interpretation has been made in respect to the N75 component, despite clearly appearing in previous TEP studies of the same brain area (Kähkönen et al., 2005; Rogasch et al., 2014). We can only hypothesize that similarly to the temporally adjacent N45, P60 and N100, the N75 also reflect GABA mediated activity (Premoli et al., 2014; Farzan et al., 2013). Further evidence on the neural significance of this component would help to determine whether this reduction in N75 following multiple treatment sessions indeed reflects an alternation of inhibitory neural activity in rPFC leading to improvement in ADHD symptoms.

Finally, our neural data provide a potentially powerful biomarker correlated with treatment efficacy. This marker is based on EEG activity recorded within the first treatment session and was found stable under both AVR and CSD. Specifically, the ratio of low Gamma to Alpha activity was correlated with ADHD symptoms improvement, accounting for 84.6% (*r* = 0.92) of variance in treatment response. As shown by the interhemispheric balance analysis, this marker has an inverse inter-hemispheric association pattern with symptomatic improvement. That is, in the rPFC it is positively associated, while in the lPFC it is negatively associated, with treatment success.

Importantly, Alpha and Gamma frequencies have been repeatedly associated with distinctive, presumably competitive, roles in terms of neural network activity in both human and animal research. Studies point that Alpha and Gamma activities co-interact with each other in an inhibitory layer specific fashion (Spaak et al., 2012). That is, bursts of Gamma activity are phase coupled with Alpha activity (Osipova et al., 2008; Spaak et al., 2012; Voytek et al., 2010), and Alpha and Gamma power are inversely correlated (Spaak et al., 2012). Indeed, greater Alpha activity is generally viewed as a hallmark of a less responsive and less functional brain state. Alpha power is inversely correlated with blood oxygenation (Laufs et al., 2003), is enhanced at rest when no input enters the visual system (Barry et al., 2007), and heightened during inhibition of task-irrelevant brain areas (Jensen and Mazaheri, 2010; Klimesch et al., 2007). Critically for the present findings, Alpha activity also reflects cortical excitability and its responsiveness to TMS. The probability of a TMS pulse to produce either a motor or a visual response is reduced with greater Alpha power under the stimulation area (Romei et al., 2008; Samaha et al., 2017; Sauseng et al., 2009). Gamma-band activity, by contrast, is positively correlated with blood oxygenation (Murta et al., 2015) and has been associated with more functional cortical state during sensory stimulation and performance of cognitive tasks (Başar, 2013). Trains of rTMS also induce long lasting (~60 s post train) elevation in both spiking rates and cortical LFP Gamma activity (Allen et al., 2007). Furthermore, Pasley et al. (2009) showed that this response pattern is dependent on the pre-train background levels of Alpha and Gamma activity (negative and positive association in accordance), thereby indicating that activation levels just prior to the stimulation delivery modulate outcome.

The current findings are consistent with the notion that the Alpha and Low-gamma activities have distinct neural origins. First, Low-gamma activity power peaks following stimulation then gradually decays, while Alpha activity power remains stable (Fig. 5A). Second, Alpha band power during treatment (at the inter-train interval) is highly correlated with that of resting state in all treatment groups, but Gamma activity is modulated by treatment (that is, resting state/treatment correlations were abolished in the Real group, attenuated in the active control group and remained high in the Sham group). Third, in line with Pasley et al. (2009), the Alpha and Low-gamma components of the biomarker form inverse association patterns with the behavioral outcome of the stimulation. Thus, it is suggested that the Low-gamma frequency observed reflects cortical response readily generated by rTMS while Alpha activity is prone to a more stable, trait-like pattern (Anokhin et al., 2006), presumably indexing cortical inhibition level (Jensen and Mazaheri, 2010). Together they mirror the responsivity of the cortex to rTMS, which is eventually reflected in behavioral and clinical outcomes. Within this model, participants whose baseline brain activity is electrophysiologically responsive to the rTMS will maximally benefit from treatment.

Notably, the neural correlates underlying the alpha component of the biomarker are in line with current knowledge of ADHD neural pathophysiology. We found a negative correlation between Alpha band power during resting state and treatment outcome at rPFC electrodes, while a positive correlation was found for lPFC electrodes (Fig. 5C). In other words, participants with less right and more left Alpha PFC activity tend to respond better to the treatment. This pattern echoes ADHD's right prefrontal hypoactivity (Aron et al., 2004, 2014; Bush et al., 2005; Dickstein et al., 2006; Pliszka et al., 2000, 2007) which is manifested, among other things, as rPFC Alpha EEG asymmetry during resting state (Hale et al., 2009, 2010; Keune et al., 2011, 2015). Within this conceptual framework, we suggest that rTMS treatment of the rPFC was more effective for participants with less profound rPFC hypoactivity.

Importantly, since most of the bandwidth of high frequency neural activity recorded using scalp surface electrodes overlaps with this of muscle activity (20–300 Hz) (Muthukumaraswamy, 2013), the low gamma component of the biomarker may be alternatively explained by residual TMS related muscles activation. Indeed, the spatial distribution of the biomarker, the lack of correlation between resting state and in-treatment low-gamma power, and the decay of power observed following stimulation trains, are in accordance with both neuronal and muscle activity. The distinction between these two alternatives is hard to make and there are competing explanations for each case. For example, greater clinical improvement can be attributed to greater intensity of stimulation (resulting from different MTs), which may induce greater neuronal effects, but also greater muscle activation. This issue should be further explored, but given future confirmation, such a biomarker, whether coming from a neuronal or a muscle source, may allow withholding treatment from those expected not to benefit, and by that to save time, money, and disappointment for many, regardless of the component's origin.

### 4.1 Study limitations

The current work was a pilot study exploring the potential benefit of a non-invasive electromagnetic stimulation treatment in ADHD participants using 3 comparison groups. As such, it suffered from several limitations. One limitation concerns the study sample which was moderate at its size, and was based on non-comorbid participants who were mostly students, thus may ill-represent the broad ADHD population. This may lead to biased results, reduced statistical power, and over-estimation of both the clinical outcome and electrophysiological biomarkers. Future studies that will only compare Real and Sham stimulation should employ larger and more representative samples in order to validate the current results. Additional limitation is the relatively high level of dropouts and discontinuation rate in the active groups compared to previous clinical trials (Carmi et al., 2019; Levkovitz et al., 2015; O'Reardon et al., 2007). This is probably due to heightened discomfort caused by the muscle contraction induced by this specific H6-coil over the target area, and perhaps given different balance between the burden of the treatment protocol and the burden of ADHD symptoms in adults, relative to the greater burden of conditions such as major depression or OCD. Future studies should determine if lower stimulation intensities may lessen dropout rates while maintaining or improving the clinical effect. Last, given the exploratory nature of the current study and although primary and secondary outcome measures were defined a-priory and we used proper statistical correction for all post-hoc and correlation analyses, it can be argued that additional correction is needed due to the use of multiple questionnaires and tasks in the Mindstreams cognitive battery. As such, future studies can use the indications obtained here to conduct a more rigorous examination of specific cognitive effects.

## 5 Conclusions

Cumulatively, this study suggests a safe, theoretically motivated, pathophysiologically relevant, non-pharmacological treatment to alleviate ADHD symptoms in adults. Furthermore, it offers a potential biomarker which can minimize physical, mental and financial burden, while maximizing the therapeutic value of the intervention. Finally, the neural and behavioral findings obtained here further establish the causative role of the rPFC in ADHD. Replication of the findings in larger samples may pave the way for a novel treatment for adult ADHD with a biomarker for patient selection.

## CRediT authorship contribution statement

**Uri Alyagon:** Methodology, Investigation, Formal analysis, Writing - original draft, Writing - review & editing. **Hamutal Shahar:** Methodology, Investigation, Project administration. **Aviad Hadar:** Writing - original draft. **Noam Barnea-Ygael:** Writing - original draft, Writing - review & editing, Visualization. **Avi Lazarovits:** Formal analysis. **Hadar Shalev:** Supervision, Investigation. **Abraham Zangen:** Conceptualization, Methodology, Writing - review & editing, Funding acquisition.

## Declaration of Competing Interest

UA, AZ filed patent PCT/IL2017/051163 entitled "Apparatus and methods for predicting therapy outcome". AZ is a consultant for, and has financial interest in, Brainsway Ltd.; a company that develops transcranial magnetic stimulation (TMS) coils designed for stimulation of deeper brain areas. UA is an EEG consultant for Brainsway Ltd.

## Appendix Supplementary materials

Image, application 1

## References

[bib0001] Allen E.A., Pasley B.N., Duong T., Freeman R.D. (2007). Transcranial magnetic stimulation elicits coupled neural and hemodynamic consequences. Science.

[bib0002] Allenby C., Falcone M., Bernardo L., Wileyto E.P., Rostain A., Ramsay J.R., Lerman C., Loughead J. (2018). Transcranial direct current brain stimulation decreases impulsivity in ADHD. Brain Stimul..

[bib0003] Amiri S., Farhang S., Ghoreishizadeh M.A., Malek A., Mohammadzadeh S. (2012). Double-blind controlled trial of venlafaxine for treatment of adults with attention deficit/hyperactivity disorder. Hum. Psychopharmacol. Clin. Exp..

[bib0004] Anokhin A.P., Heath A.C., Myers E. (2006). Genetic and environmental influences on frontal EEG asymmetry: a twin study. Biol. Psychol..

[bib0005] Arns M., Gunkelman J., Breteler M., Spronk D. (2008). Eeg phenotypes predict treatment outcome to stimulants in children with adhd. J. Integr. Neurosci..

[bib0006] Aron A.R., Robbins T.W., Poldrack R.A. (2014). Inhibition and the right inferior frontal cortex: one decade on. Trends Cognit. Sci..

[bib0007] Aron A.R., Robbins T.W., Poldrack R.A. (2004). Inhibition and the right inferior frontal cortex. Trends Cognit. Sci..

[bib0008] Bandeira I.D., Guimarães R.S.Q., Jagersbacher J.G., Barretto T.L., de Jesus-Silva J.R., Santos S.N., Argollo N., Lucena R. (2016). Transcranial direct current stimulation in children and adolescents with attention-deficit/hyperactivity disorder (ADHD): a pilot study. J. Child Neurol..

[bib0009] Barkley R.A. (2010). Adult ADHD Rating Scale - IV.

[bib0010] Barry R.J., Clarke A.R., Johnstone S.J., Magee C.A., Rushby J.A. (2007). EEG differences between eyes-closed and eyes-open resting conditions. Clin. Neurophysiol..

[bib0011] Başar E. (2013). A review of gamma oscillations in healthy subjects and in cognitive impairment. Int. J. Psychophysiol..

[bib0012] Beaulieu L.-D., Milot M.-H. (2018). Changes in transcranial magnetic stimulation outcome measures in response to upper-limb physical training in stroke: a systematic review of randomized controlled trials. Ann. Phys. Rehabil. Med..

[bib0013] Beck A.T., Ward C.H., Mendelson M., Mock J., Erbaugh J. (1961). An inventory for measuring depression. Arch. Gen. Psychiatry.

[bib0014] Benadhira R., Thomas F., Bouaziz N., Braha S., Andrianisaina P.S.-K., Isaac C., Moulier V., Januel D. (2017). A randomized, sham-controlled study of maintenance rTMS for treatment-resistant depression (TRD). Psychiatry Res..

[bib0015] Benjamini Y., Hochberg Y. (1995). Controlling the false discovery rate: a practical and powerful approach to multiple testing. J. R. Stat. Soc. Ser. B Methodol..

[bib0016] Biederman J., Spencer T., Wilens T. (2004). Evidence-based pharmacotherapy for attention-deficit hyperactivity disorder. Int. J. Neuropsychopharmacol..

[bib0017] Bloch Y., Harel E.V., Aviram S., Govezensky J., Ratzoni G., Levkovitz Y. (2010). Positive effects of repetitive transcranial magnetic stimulation on attention in ADHD subjects: a randomized controlled pilot study. World J. Biol. Psychiatry.

[bib0018] Breitling C., Zaehle T., Dannhauer M., Bonath B., Tegelbeckers J., Flechtner H.-H., Krauel K. (2016). Improving Interference control in ADHD patients with transcranial direct current stimulation (tDCS). Front. Cell. Neurosci..

[bib0019] Bush G., Valera E.M., Seidman L.J. (2005). Functional neuroimaging of attention-deficit/hyperactivity disorder: a review and suggested future directions. Biol. Psychiatry.

[bib0020] Cachoeira C.T., Leffa D.T., Mittelstadt S.D., Mendes L.S.T., Brunoni A.R., Pinto J.V., Blazius V., Machado V., Dotto Bau C.H., Rohde L.A., Grevet E.H., Schestatsky P. (2017). Positive effects of transcranial direct current stimulation in adult patients with attention-deficit/hyperactivity disorder – a pilot randomized controlled study. Psychiatry Res..

[bib0021] Cao P., Wang L., Cheng Q., Sun X., Kang Q., Dai L., Zhou X., Song Z. (2019). Changes in serum miRNA-let-7 level in children with attention deficit hyperactivity disorder treated by repetitive transcranial magnetic stimulation or atomoxetine: an exploratory trial. Psychiatry Res..

[bib0022] Cao P., Xing J., Cao Y., Cheng Q., Sun X., Kang Q., Dai L., Zhou X., Song Z. (2018). Clinical effects of repetitive transcranial magnetic stimulation combined with atomoxetine in the treatment of attention-deficit hyperactivity disorder. Neuropsychiatr. Dis. Treat..

[bib0023] Carmi L., Alyagon U., Barnea-Ygael N., Zohar J., Dar R., Zangen A. (2017). Clinical and electrophysiological outcomes of deep TMS over the medial prefrontal and anterior cingulate cortices in OCD patients. Brain Stimul..

[bib0024] Carmi L., Tendler A., Bystritsky A., Hollander E., Blumberger D.M., Daskalakis J., Ward H., Lapidus K., Goodman W., Casuto L., Feifel D., Barnea-Ygael N., Roth Y., Zangen A., Zohar J. (2019). Efficacy and safety of deep transcranial magnetic stimulation for obsessive-compulsive disorder: a prospective multicenter randomized double-blind placebo-controlled trial. Am. J. Psychiatry.

[bib0025] Cheng J.Y.W., Chen R.Y.L., Ko J.S.N., Ng E.M.L. (2007). Efficacy and safety of atomoxetine for attention-deficit/hyperactivity disorder in children and adolescents—meta-analysis and meta-regression analysis. Psychopharmacology.

[bib0026] Conners C.K., Erhardt D., Epstein J.N., Parker J.D.A., Sitarenios G., Sparrow E. (1999). Self-ratings of ADHD symptoms in adults I: factor structure and normative data. J. Atten. Disord..

[bib0027] Conners C.K., Erhardt D., Sparrow E. (1999). CAARS: Technical Manual.

[bib0028] Cosmo C., Baptista A.F., Araújo A.N.de, Rosário R.S.do, Miranda J.G.V., Montoya P., Sena E.P.de (2015). A randomized, double-blind, sham-controlled trial of transcranial direct current stimulation in attention-deficit/hyperactivity disorder. PLoS ONE.

[bib0029] Delorme A., Makeig S. (2004). EEGLAB: an open source toolbox for analysis of single-trial EEG dynamics including independent component analysis. J. Neurosci. Methods.

[bib0030] Dickstein S.G., Bannon K., Xavier Castellanos F., Milham M.P. (2006). The neural correlates of attention deficit hyperactivity disorder: an ALE meta-analysis. J. Child Psychol. Psychiatry.

[bib0031] Dinteren R., Arns M., Kenemans L., Jongsma M.L.A., Kessels R.P.C., Fitzgerald P., Fallahpour K., Debattista C., Gordon E., Williams L.M. (2015). Utility of event-related potentials in predicting antidepressant treatment response: an iSPOT-D report. Eur. Neuropsychopharmacol..

[bib0032] Dinur-Klein L., Dannon P., Hadar A., Rosenberg O., Roth Y., Kotler M., Zangen A. (2014). Smoking cessation induced by deep repetitive transcranial magnetic stimulation of the prefrontal and insular cortices: a prospective, randomized controlled trial. Biol. Psychiatry Alcohol. Smok..

[bib0033] Doniger G.M. (2008). Mindstreams: Guide to Normative Data.

[bib0034] Durell T.M., Adler L.A., Williams D.W., Deldar A., McGough J.J., Glaser P.E., Rubin R.L., Pigott T.A., Sarkis E.H., Fox B.K. (2013). Atomoxetine treatment of attention-deficit/hyperactivity disorder in young adults with assessment of functional outcomes: a randomized, double-blind, placebo-controlled clinical trial. J. Clin. Psychopharmacol..

[bib0035] Dwolatzky T., Whitehead V., Doniger G.M., Simon E.S., Schweiger A., Jaffe D., Chertkow H. (2003). Validity of a novel computerized cognitive battery for mild cognitive impairment. BMC Geriatr..

[bib0036] Erhardt D., Epstein J.N., Conners C.K., Parker J.D.A., Sitarenios G. (1999). Self-ratings of ADHD symptomas in adults II: reliability, validity, and diagnostic sensitivity. J. Atten. Disord..

[bib0037] Faraone S.V., Biederman J. (2002). Efficacy of Adderall® for attention-deficit/hyperactivity disorder: a meta-analysis. J. Atten. Disord..

[bib0038] Faraone S.V., Spencer T., Aleardi M., Pagano C., Biederman J. (2004). Meta-analysis of the efficacy of methylphenidate for treating adult attention-deficit/hyperactivity disorder. J. Clin. Psychopharmacol..

[bib0039] Farzan F., Barr M.S., Hoppenbrouwers S.S., Fitzgerald P.B., Chen R., Pascual-Leone A., Daskalakis Z.J. (2013). The EEG correlates of the TMS-induced EMG silent period in humans. NeuroImage.

[bib0040] Fayyad J., Graaf R.D., Kessler R., Alonso J., Angermeyer M., Demyttenaere K., Girolamo G.D., Haro J.M., Karam E.G., Lara C., Lépine J.-P., Ormel J., Posada-Villa J., Zaslavsky A.M., Jin R. (2007). Cross–national prevalence and correlates of adult attention–deficit hyperactivity disorder. Br. J. Psychiatry.

[bib0041] Fitzgerald P.B., Fountain S., Daskalakis Z.J. (2006). A comprehensive review of the effects of rTMS on motor cortical excitability and inhibition. Clin. Neurophysiol..

[bib0042] Fitzgerald P.B., Maller J.J., Hoy K.E., Thomson R., Daskalakis Z.J. (2009). Exploring the optimal site for the localization of dorsolateral prefrontal cortex in brain stimulation experiments. Brain Stimul..

[bib0043] Gómez L., Vidal B., Morales L., Báez M., Maragoto C., Galvizu R., Vera H., Cabrera I., Zaldívar M., Sánchez A. (2014). Low frequency repetitive transcranial magnetic stimulation in children with attention deficit/hyperactivity disorder. Preliminary results. Brain Stimul..

[bib0044] Hagemann D. (2004). Individual differences in anterior EEG asymmetry: methodological problems and solutions. Biol. Psychol..

[bib0045] Hagemann D., Naumann E., Thayer J.F. (2001). The quest for the EEG reference revisited: a glance from brain asymmetry research. Psychophysiology.

[bib0046] Hale T.S., Smalley S.L., Dang J., Hanada G., Macion J., McCracken J.T., McGough J.J., Loo S.K. (2010). ADHD familial loading and abnormal EEG alpha asymmetry in children with ADHD. J. Psychiatr. Res..

[bib0047] Hale T.S., Smalley S.L., Hanada G., Macion J., McCracken J.T., McGough J.J., Loo S.K. (2009). Atypical alpha asymmetry in adults with ADHD. Neuropsychologia.

[bib0048] Hart H., Radua J., Nakao T., Mataix-Cols D., Rubia K. (2013). Meta-analysis of functional magnetic resonance imaging studies of inhibition and attention in attention-deficit/hyperactivity disorder: exploring task-specific, stimulant medication, and age effects. JAMA Psychiatry.

[bib0049] Isserles M., Shalev A.Y., Roth Y., Peri T., Kutz I., Zlotnick E., Zangen A. (2013). Effectiveness of deep transcranial magnetic stimulation combined with a brief exposure procedure in post-traumatic stress disorder – a pilot study. Brain Stimul..

[bib0050] Jacoby N., Lavidor M. (2018). Null tDCS effects in a sustained attention task: the modulating role of learning. Front. Psychol..

[bib0051] Jensen O., Mazaheri A. (2010). Shaping functional architecture by oscillatory alpha activity: gating by inhibition. Front. Hum. Neurosci..

[bib0052] Jones B., Jarvis P., Lewis J.A., Ebbutt A.F. (1996). Trials to assess equivalence: the importance of rigorous methods. BMJ.

[bib0053] Kähkönen S., Komssi S., Wilenius J., Ilmoniemi R.J. (2005). Prefrontal transcranial magnetic stimulation produces intensity-dependent EEG responses in humans. NeuroImage.

[bib0054] Keune P.M., Schönenberg M., Wyckoff S., Mayer K., Riemann S., Hautzinger M., Strehl U. (2011). Frontal alpha-asymmetry in adults with attention deficit hyperactivity disorder: replication and specification. Biol. Psychol..

[bib0055] Keune P.M., Wiedemann E., Schneidt A., Schönenberg M. (2015). Frontal brain asymmetry in adult attention-deficit/hyperactivity disorder (ADHD): extending the motivational dysfunction hypothesis. Clin. Neurophysiol..

[bib0056] Klimesch W., Sauseng P., Hanslmayr S. (2007). EEG alpha oscillations: the inhibition–timing hypothesis. Brain Res. Rev..

[bib0057] Koch G., Bonnì S., Pellicciari M.C., Casula E.P., Mancini M., Esposito R., Ponzo V., Picazio S., Di Lorenzo F., Serra L., Motta C., Maiella M., Marra C., Cercignani M., Martorana A., Caltagirone C., Bozzali M. (2018). Transcranial magnetic stimulation of the precuneus enhances memory and neural activity in prodromal Alzheimer's disease. NeuroImage.

[bib0058] Laufs H., Kleinschmidt A., Beyerle A., Eger E., Salek-Haddadi A., Preibisch C., Krakow K. (2003). EEG-correlated fMRI of human alpha activity. NeuroImage.

[bib0059] Lefaucheur J.-P., André-Obadia N., Antal A., Ayache S.S., Baeken C., Benninger D.H., Cantello R.M., Cincotta M., de Carvalho M., De Ridder D., Devanne H., Di Lazzaro V., Filipović S.R., Hummel F.C., Jääskeläinen S.K., Kimiskidis V.K., Koch G., Langguth B., Nyffeler T., Oliviero A., Padberg F., Poulet E., Rossi S., Rossini P.M., Rothwell J.C., Schönfeldt-Lecuona C., Siebner H.R., Slotema C.W., Stagg C.J., Valls-Sole J., Ziemann U., Paulus W., Garcia-Larrea L. (2014). Evidence-based guidelines on the therapeutic use of repetitive transcranial magnetic stimulation (rTMS). Clin. Neurophysiol..

[bib0060] Levkovitz Y., Isserles M., Padberg F., Lisanby S.H., Bystritsky A., Xia G., Tendler A., Daskalakis Z.J., Winston J.L., Dannon P., Hafez H.M., Reti I.M., Morales O.G., Schlaepfer T.E., Hollander E., Berman J.A., Husain M.M., Sofer U., Stein A., Adler S., Deutsch L., Deutsch F., Roth Y., George M.S., Zangen A. (2015). Efficacy and safety of deep transcranial magnetic stimulation for major depression: a prospective multicenter randomized controlled trial. World Psychiatry.

[bib0061] Lieshout E.C.C.van, Visser-Meily J.M.A., Neggers S.F.W., Worp H.B.van der, Dijkhuizen R.M. (2017). Brain stimulation for arm recovery after stroke (B-STARS): protocol for a randomised controlled trial in subacute stroke patients. BMJ Open.

[bib0062] Montoya A., Quail D., Anand E., Cardo E., Alda J.A., Escobar R. (2014). Prognostic factors of improvement in health-related quality of life in atomoxetine-treated children and adolescents with attention-deficit/hyperactivity disorder, based on a pooled analysis. ADHD Atten. Deficit Hyperact. Disord..

[bib0063] Mottaghy F.M., Gangitano M., Sparing R., Krause B.J., Pascual-Leone A. (2002). Segregation of areas related to visual working memory in the prefrontal cortex revealed by rTMS. Cereb. Cortex.

[bib0064] Murta T., Leite M., Carmichael D.W., Figueiredo P., Lemieux L. (2015). Electrophysiological correlates of the BOLD signal for EEG-informed fMRI. Hum. Brain Mapp..

[bib0065] Muthukumaraswamy S.D. (2013). High-frequency brain activity and muscle artifacts in MEG/EEG: a review and recommendations. Front. Hum. Neurosci..

[bib0066] Nejati V., Salehinejad M.A., Nitsche M.A., Najian A., Javadi A.-H. (2017). Transcranial direct current stimulation improves executive dysfunctions in ADHD: implications for inhibitory control, interference control, working memory, and cognitive flexibility. J. Atten. Disord..

[bib0067] Newcorn J.H., Sutton V.K., Zhang S., Wilens T., Kratochvil C., Emslie G.J., D'souza D.N., Schuh L.M., Allen A.J. (2009). Characteristics of placebo responders in pediatric clinical trials of attention-deficit/hyperactivity disorder. J. Am. Acad. Child Adolesc. Psychiatry.

[bib0068] Niederhofer H. (2012). Additional biological therapies for attention-deficit hyperactivity disorder: repetitive transcranical magnetic stimulation of 1 Hz helps to reduce methylphenidate. Clin. Pract..

[bib0069] Niederhofer H. (2008). Effectiveness of the repetitive transcranical magnetic stimulation (rTMS) of 1 Hz for attention-deficit hyperactivity disorder (ADHD). Psychiatr. Danub..

[bib0070] Noda Y., Zomorrodi R., Cash R.F.H., Barr M.S., Farzan F., Rajji T.K., Chen R., Daskalakis Z.J., Blumberger D.M. (2017). Characterization of the influence of age on GABAA and glutamatergic mediated functions in the dorsolateral prefrontal cortex using paired-pulse TMS-EEG. Aging.

[bib0071] Norman L.J., Carlisi C., Lukito S., Hart H., Mataix-Cols D., Radua J., Rubia K. (2016). Structural and functional brain abnormalities in attention-deficit/hyperactivity disorder and obsessive-compulsive disorder: a comparative meta-analysis. JAMA Psychiatry.

[bib0072] Oostenveld R., Fries P., Maris E., Schoffelen J.-M. (2011). FieldTrip: open source software for advanced analysis of MEG, EEG, and invasive electrophysiological data. Intell. Neurosci..

[bib0073] O’Reardon J.P., Solvason H.B., Janicak P.G., Sampson S., Isenberg K.E., Nahas Z., McDonald W.M., Avery D., Fitzgerald P.B., Loo C., Demitrack M.A., George M.S., Sackeim H.A. (2007). Efficacy and safety of transcranial magnetic stimulation in the acute treatment of major depression: a multisite randomized controlled trial. Biol. Psychiatry Depress..

[bib0074] Osipova D., Hermes D., Jensen O. (2008). Gamma power is phase-locked to posterior alpha activity. PLoS ONE.

[bib0075] Osman A., Lou L., Muller-Gethmann H., Rinkenauer G., Mattes S., Ulrich R. (2000). Mechanisms of speed–accuracy tradeoff: evidence from covert motor processes. Biol. Psychol..

[bib0076] Pascual-Leone A., Amedi A., Fregni F., Merabet L.B. (2005). The plastic human brain cortex. Annu. Rev. Neurosci..

[bib0077] Pasley B.N., Allen E.A., Freeman R.D. (2009). State-dependent variability of neuronal responses to transcranial magnetic stimulation of the visual cortex. Neuron.

[bib0078] Paz Y., Friedwald K., Levkovitz Y., Zangen A., Alyagon U., Nitzan U., Segev A., Maoz H., Koubi M., Bloch Y. (2017). Randomised sham-controlled study of high-frequency bilateral deep transcranial magnetic stimulation (dTMS) to treat adult attention hyperactive disorder (ADHD): negative results. World J. Biol. Psychiatry.

[bib0079] Pliszka S.R., Liotti M., Bailey B.Y., Perez III R., Glahn D., Semrud-Clikeman M. (2007). Electrophysiological effects of stimulant treatment on inhibitory control in children with attention-deficit/hyperactivity disorder. J. Child Adolesc. Psychopharmacol..

[bib0080] Pliszka S.R., Liotti M., Woldorff M.G. (2000). Inhibitory control in children with attention-deficit/hyperactivity disorder: event-related potentials identify the processing component and timing of an impaired right-frontal response-inhibition mechanism. Biol. Psychiatry.

[bib0081] Premoli I., Castellanos N., Rivolta D., Belardinelli P., Bajo R., Zipser C., Espenhahn S., Heidegger T., Müller-Dahlhaus F., Ziemann U. (2014). TMS-EEG signatures of GABAergic neurotransmission in the human cortex. J. Neurosci..

[bib0082] Richieri R., Guedj E., Michel P., Loundou A., Auquier P., Lançon C., Boyer L. (2013). Maintenance transcranial magnetic stimulation reduces depression relapse: a propensity-adjusted analysis. J. Affect. Disord..

[bib0083] Rogasch N.C., Fitzgerald P.B. (2013). Assessing cortical network properties using TMS–EEG. Hum. Brain Mapp..

[bib0084] Rogasch N.C., Thomson R.H., Farzan F., Fitzgibbon B.M., Bailey N.W., Hernandez-Pavon J.C., Daskalakis Z.J., Fitzgerald P.B. (2014). Removing artefacts from TMS-EEG recordings using independent component analysis: importance for assessing prefrontal and motor cortex network properties. NeuroImage.

[bib0085] Romei V., Rihs T., Brodbeck V., Thut G. (2008). Resting electroencephalogram alpha-power over posterior sites indexes baseline visual cortex excitability. Neuroreport.

[bib0086] Roth R.M., Isquith P.M., Gioia G.A. (2005). Behavior Rating Inventory of Executive Function: Adult Version.

[bib0087] Roth Y., Amir A., Levkovitz Y., Zangen A. (2007). Three-dimensional distribution of the electric field induced in the brain by transcranial magnetic stimulation using figure-8 and deep H-coils. J. Clin. Neurophysiol..

[bib0088] Roth Y., Zangen A., Rotenberg A., Horvath J.C., Pascual-Leone A. (2014). Reaching deep brain structures: the H-coils. Transcranial Magnetic Stimulation, Neuromethods.

[bib0089] Roth Y., Zangen A., Hallett M. (2002). A coil design for transcranial magnetic stimulation of deep brain regions. J. Clin. Neurophysiol..

[bib0090] Rubia K., Alegría A.A., Brinson H. (2014). Brain abnormalities in attention-deficit hyperactivity disorder: a review. Rev. Neurol..

[bib0091] Salehinejad M.A., Wischnewski M., Nejati V., Vicario C.M., Nitsche M.A. (2019). Transcranial direct current stimulation in attention-deficit hyperactivity disorder: a meta-analysis of neuropsychological deficits. PLoS ONE.

[bib0092] Samaha J., Gosseries O., Postle B.R. (2017). Distinct oscillatory frequencies underlie excitability of human occipital and parietal cortex. J. Neurosci..

[bib0093] Samea F., Soluki S., Nejati V., Zarei M., Cortese S., Eickhoff S.B., Tahmasian M., Eickhoff C.R. (2019). Brain alterations in children/adolescents with ADHD revisited: a neuroimaging meta-analysis of 96 structural and functional studies. Neurosci. Biobehav. Rev..

[bib0094] Sauseng P., Klimesch W., Gerloff C., Hummel F.C. (2009). Spontaneous locally restricted EEG alpha activity determines cortical excitability in the motor cortex. Neuropsychologia.

[bib0095] Schachter H.M., Pham B., King J., Langford S., Moher D. (2001). How efficacious and safe is short-acting methylphenidate for the treatment of attention-deficit disorder in children and adolescents? A meta-analysis. Can. Med. Assoc. J..

[bib0096] Schweiger A., Abramovitch A., Doniger G.M., Simon E.S. (2007). A clinical construct validity study of a novel computerized battery for the diagnosis of ADHD in young adults. J. Clin. Exp. Neuropsychol..

[bib0097] Schweiger A., Doniger G., Dwolatzky T., Jaffe D., Simon E. (2003). Reliability of a novel computerized neuropsychological battery for mild cognitive impairment. Acta Neuropsychol..

[bib0098] Silberstein R.B., Levy F., Pipingas A., Farrow M. (2017). First-dose methylphenidate–induced changes in brain functional connectivity are correlated with 3-month attention-deficit/hyperactivity disorder symptom sesponse. Biol. Psychiatry.

[bib0099] Soff C., Sotnikova A., Christiansen H., Becker K., Siniatchkin M. (2017). Transcranial direct current stimulation improves clinical symptoms in adolescents with attention deficit hyperactivity disorder. J. Neural Transm..

[bib0100] Soltaninejad Z., Nejati V., Ekhtiari H. (2019). Effect of anodal and cathodal transcranial direct current stimulation on DLPFC on modulation of inhibitory control in ADHD. J. Atten. Disord..

[bib0101] Sonuga-Barke E.J.S. (2005). Causal models of attention-deficit/hyperactivity disorder: from common simple deficits to multiple developmental pathways. Biol. Psychiatry.

[bib0102] Sotnikova A., Soff C., Tagliazucchi E., Becker K., Siniatchkin M. (2017). Transcranial direct current stimulation modulates neuronal networks in attention deficit hyperactivity disorder. Brain Topogr..

[bib0103] Spaak E., Bonnefond M., Maier A., Leopold D.A., Jensen O. (2012). Layer-specific entrainment of gamma-band neural activity by the alpha rhythm in monkey visual cortex. Curr. Biol..

[bib0104] Stern A., Malik E., Pollak Y., Bonne O., Maeir A. (2016). The efficacy of computerized cognitive training in adults with ADHD: a randomized controlled trial. J. Atten. Disord..

[bib0105] Sun Y., Farzan F., Mulsant B.H., Rajji T.K., Fitzgerald P.B., Barr M.S., Downar J., Wong W., Blumberger D.M., Daskalakis Z.J. (2016). Indicators for remission of suicidal ideation following magnetic seizure therapy in patients with treatment-resistant depression. JAMA Psychiatry.

[bib0106] Tenke C.E., Kayser J., Manna C.G., Fekri S., Kroppmann C.J., Schaller J.D., Alschuler D.M., Stewart J.W., McGrath P.J., Bruder G.E. (2011). Current source density measures of electroencephalographic alpha predict antidepressant treatment response. Biol. Psychiatry.

[bib0107] Thomas R., Sanders S., Doust J., Beller E., Glasziou P. (2015). Prevalence of attention-deficit/hyperactivity disorder: a systematic review and meta-analysis. Pediatrics.

[bib0108] Vanneste S., Ridder D.D. (2012). The involvement of the left ventrolateral prefrontal cortex in tinnitus: a TMS study. Exp. Brain Res..

[bib0109] Voytek B., Canolty R.T., Shestyuk A., Crone N.E., Parvizi J., Knight R.T. (2010). Shifts in gamma phase–amplitude coupling frequency from theta to alpha over posterior cortex during visual tasks. Front. Hum. Neurosci..

[bib0110] Weaver L., Rostain A.L., Mace W., Akhtar U., Moss E., O'Reardon J.P. (2012). Transcranial magnetic stimulation (TMS) in the treatment of attention-deficit/hyperactivity disorder in adolescents and young adults: a pilot study. J. ECT.

[bib0111] Wheeler R.E., Davidson R.J., Tomarken A.J. (1993). Frontal brain asymmetry and emotional reactivity: a biological substrate of affective style. Psychophysiology.

[bib0112] Willcutt E.G., Doyle A.E., Nigg J.T., Faraone S.V., Pennington B.F. (2005). Validity of the executive function theory of attention-deficit/hyperactivity disorder: a meta-analytic review. Biol. Psychiatry.

[bib0113] Zangen A., Roth Y., Voller B., Hallett M. (2005). Transcranial magnetic stimulation of deep brain regions: evidence for efficacy of the H-coil. Clin. Neurophysiol..

