# Supplementary material for: Alleviation of ADHD symptoms by non-invasive right prefrontal stimulation is correlated with EEG activity
Source: Neuroimage Clin. 2020 Feb 6;26:102206. doi: 10.1016/j.nicl.2020.102206 (PMC7021642; doi:10.1016/j.nicl.2020.102206)
Supplement: Supplementary file 1 [file mmc1.docx]

**Supplementary methods and materials**

*Stop signal task*

A modified version of the visual stop signal task was presented on a lab PC using Eprime (PST Inc., E-Prime 2.0). The task presented two visual cues (‘x’, ‘o’) to which participants had to respond by pressing one of two corresponding buttons using either their left or right index fingers. In 25% of trials, a stop signal (white square) was presented immediately after the visual cue, instructing the participants to withhold their response. The stop signal delay from visual cue onset changed in a staircase dynamic-tracking manner, depending on performance (Berger et al., 2013; Hadar et al., 2017). The Stop Signal Reaction Time was computed as subtraction of the mean delay of the stop trials from the mean reaction time of the go trials.

*EEG recording and preprocessing methods*

The EEG was acquired using a TMS-compatible 64-channel amplifier and cap (ANT Ltd.) with Cz as a common reference (re-referenced offline to the average) and PO6 as ground. Impedance was kept below 10 kOhm and signal was digitized at 2048 Hz using a 24-bit AD converter.

Preprocessing was conducted using EEGlab (Delorme and Makeig, 2004) in Matlab. TEP (TMS Evoked Potentials) data from the first and last sessions (D1 and D15) was segmented around the TMS pulse (1 second before to 1 second after the pulse) and baseline corrected (200 to 10 ms before pulse). Data from the first 40 ms post pulse EEG response (-1 to 40 ms), which contains a massive electrical artifact as well as dominant lateral muscle activation, was deleted. This is a fairly wide deletion compared to other methods but was favored because it excludes the lateral muscle artifact almost completely, obviates the need to perform two distinct ICA (Independent Components Analysis) decompositions (Rogasch et al., 2014), and makes the artifact removal process less ambiguous (note the small number of removed components compare to Rogasch et al. (Rogasch et al., 2014)). The data was then filtered (1-48 Hz bandpass FIR (Finite Impulse Response) filter; EEGlab function pop_eegfiltnew) and manually scanned for segments containing excessive TMS-related artifacts, which will jeopardize the integrity of the ICA decomposition. Afterwards, an infomax ICA decomposition was executed (EEGlab function pop_runica) and TMS related components were identified and cleared semi-automatically using the TMS-EEG signal analyzer plugin (Rogasch et al., 2017) (TESA, number of cleared components: M= 2.76, sd=2.71). Additional automatic artifact detection was then conducted, clearing deviant (3 SD or higher from the mean entropy value) channels or epochs contaminated with residual artifacts (EEGlab function pop_rejprob; number of excluded epochs per file: M=9.77, sd=3.95; number of excluded channels per file: M=6, sd=3.22).

Treatment data was extracted from the Inter-train intervals (ITI), filtered (1-100 Hz bandpass, 48-52 Hz notch FIR filter) and segmented into 2 seconds epochs starting 1 second after train's ending (to avoid TMS related artifacts induced by the stimulation) and ceasing 5 seconds before the upcoming train (to avoid activity alterations caused by anticipation to the next train; total of 7 segments per ITI). Data was then manually scanned and noisy channels or epochs containing residual TMS related artifacts were excluded. Eye movements were removed using ICA and power spectral density was then computed (EEGlab function pop_spectopo).

Resting state EEG was recorded for 180 seconds (30 initial seconds were truncated to prevent state transitional influence). The pre-processing of the resting state data was similar to that of the treatment, except that epochs containing excessive eye-movements related low frequency activity (2-4 Hz), or muscle related high frequency noise (20-40 Hz), were rejected (using EEGlab function pop_rejspec and subsequent manual inspection. Number of excluded epochs per file: M=10.05, sd=7.54; number of excluded channels per file: M= 0.26, sd=0.6). This strategy is preferred over ICA components' deletion when data is abundant (Jung et al., 2000).

*Additional statistical methods*

Correlations between the clinical primary outcome measure and the behavioral scores in the Minstreams cognitive battery were computed using Pearson linear coefficient. Significance values were Bonferroni corrected for 18 tests (6 cognitive scores X 3 groups). An erratic association pattern and amplified within-group variance were observed in the AC group (and not in Sham group where there is no correlation but observations are nicely clustered; see Fig. 2B, C). Those raised concerns that individual differences in brain structure of participants, together with the relatively narrow stimulation area of the Figure-8 coil, resulted in variable cognitive outcomes which inflated the statistical error term specifically for the AC group. Therefore, we executed separate analysis of variance to compare the change in Stroop performance of the Real group to the Sham and AC groups.

Stimulation related changes in marker's activity were tested using non-parametric permutation analysis implemented in FieldTrip (Maris and Oostenveld, 2007; Oostenveld et al., 2011) (Monte-Carlo method ; independent t test statistics) comparing active stimulation groups and the Sham group.

Correlations between treatment and resting state activity were calculated based on the data from the first segment of the ITI during the first treatment and the resting state activity (just prior to the treatment and before any stimulation had been delivered).

In order to explore brain activity dynamics during the ITI of the first treatment session, we first computed the power ratios in the Alpha and Low-gamma bands between activity in each segment of the ITI (6 segments, seconds 3-15 after train's ending) and the initial post train segment (seconds 1-3). Those measures were then analyzed using two-way repeated measures ANOVA with frequency (Alpha, Low-gamma) and ITI time (6 segments). Post hoc comparisons were Bonferroni corrected.

Treatment effects upon TEP were tested using permutation analysis in the 5 time windows of interest (TOIs) as were observed in the data and in accordance with previous literature (Kähkönen et al., 2005; Premoli et al., 2014; Rogasch et al., 2014) (N45 : 40-50ms; P60 : 55-65ms; N75 : 70-80; N100 : 110-130ms; P180 :160-240ms). Time X group interactions were tested in the following manner: difference averaged waves (D15-D1) were first computed for each subject. Then, the mean difference amplitudes in the TOI were subjected to a between groups cluster-based permutation test using F statistics. Following the logic of interaction testing in regular parametric ANOVA, specific contrasts between the Real and the Sham/AC groups were investigated only if time X group interaction identified a significant cluster.

Treatment effects upon resting state activity power were tested in the same manner as the TEP (i.e. time X group interactions using a cluster based permutation test with F statistics) in 5 frequency bands: Delta (1-3.5 Hz), Theta (4-7.5 Hz), Alpha (8-14.5 Hz), Beta (15-29.5 Hz) and Low-gamma (30-40 Hz).

*Seizure case report*

A university student male patient (age 23) was recruited for the study and assign to the AC group. He received 2 treatments without reporting any side effects but underwent a tonic-clonic seizure during the third. Motor threshold was checked before treatment (MT was 65%, and stimulation intensity 78%, of the stimulator output). The patient was examined in the local emergency room and released after few hours. He reported sleeping below average in the night before treatment.

*Treatment influence is not mediated by depression level alternations*

To investigate if treatment influence could be explained by alternations in depression level caused by the rTMS stimulation, we conducted an additional analysis including BDI improvement as a covariant in the primary outcome measure ANOVA. No effect of BDI improvement on CAARS total ADHD scores was found (F(1,37)=0.13, n.s), the Time X Group interaction was marginally significant (F(2,37)=2.66, p=0.08; η2p=0.12), and post-hoc analysis still revealed a significant improvement in the Real group but not in the other groups (F(1,37)=17.81, p_c_=0.00015; η2p=0.32).

**Table S1. Number of participants included in each analysis.**

|  | Real | AC | Sham |  |
| --- | --- | --- | --- | --- |
| Treated | 15 | 14 | 14 |  |
| CAARS-ANOVA primary endpoint | 15 | 13 | 14 | Deviant score (1 subject) |
| CAARS – response rate primary endpoint | 15 | 14 | 14 |  |
| CAARS-ANOVA follow-up | 11 | 11 | 13 | Deviant score (1 subject) |
| CAARS – response rate follow-up | 11 | 12 | 13 |  |
| Stroop – ANOVA and correlations | 15 | 13 | 13 | Failed to complete a color detection pre-check (1); Did not attend the cognitive check (1) |
| TEP | 13 | 12 | 13 | Insufficient data quality (5) |
| Treatment EEG – correlation model | 15 | 12 | 14 | Insufficient data quality (2) |
| Resting state EEG – correlation model | 15 |  |  |  |
| ITI dynamics | 19 |  |  | Insufficient data quality (1) |
| Treatment – Resting state EEG correlations | 19 | 15 | 15 | Insufficient data quality (3) |

AC – Active Control.

**
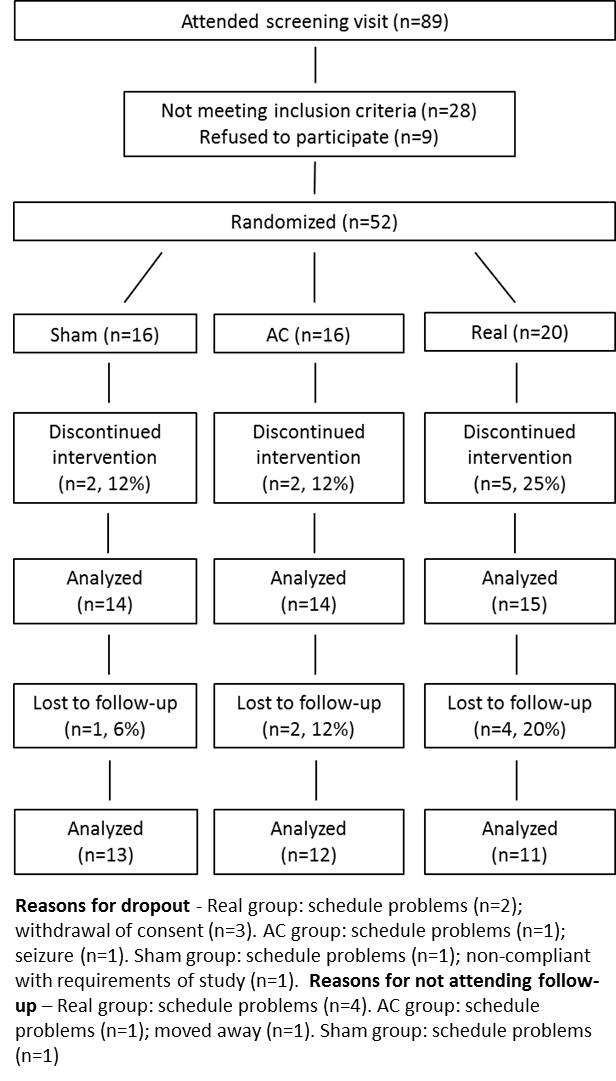
**

**Fig. S1. CONSORT diagram**. AC – Active Control

**
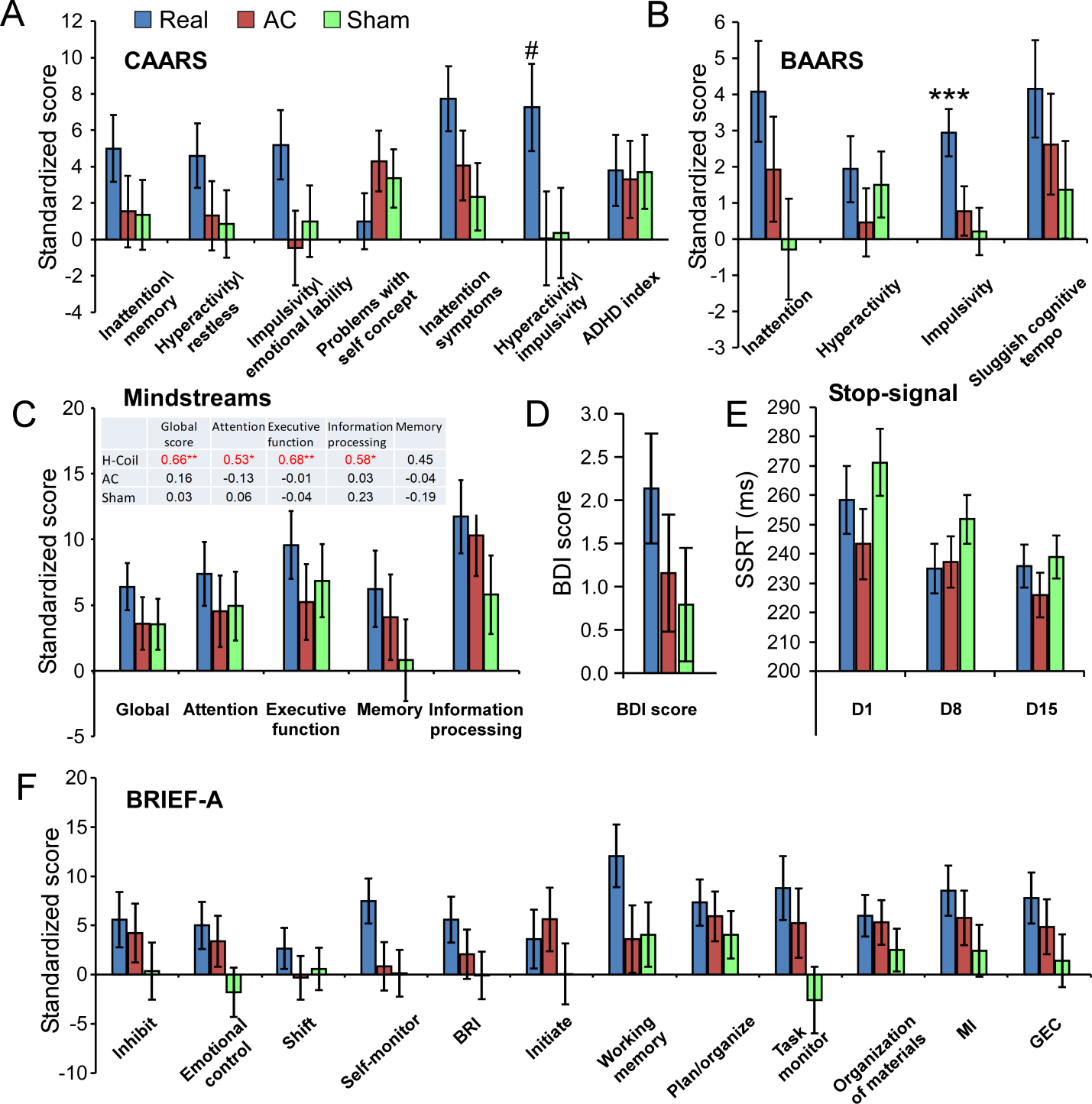
**

**Fig. S2**. **Secondary clinical and behavioral measures**. Improvement in the clinical secondary measures are shown for the subscales of (**A**) CAARS (**B**) BAARS (**C**) ADHD related cognitive domains from the Mindstreams cognitive battery, including an inset of correlations with clinical improvement. (**D**) BDI (**E**) Stop Signal Task. Mean group stop signal reaction times are shown for the Pre, middle-, and post-treatment time-points. (**F**) BRIEF-A. AC – Active Control; CAARS - Conners’ Adult ADHD Rating Scale; BAARS - Barkely Adult ADHD Rating Scale – IV; BDI - Beck Depression Inventory; BRIEF – A - Behavioral Rating Inventory for Executive Functioning; BRI – Behavioral Regulation Index; MI – Meta cognition Index; GEC – Global Executive Composite. ***p_interaction_=0.001 and p_c_=0.001; ^#^p_interaction_=0.075 and p_c_=0.065.

**Table S2 Detailed statistical results of secondary measures**


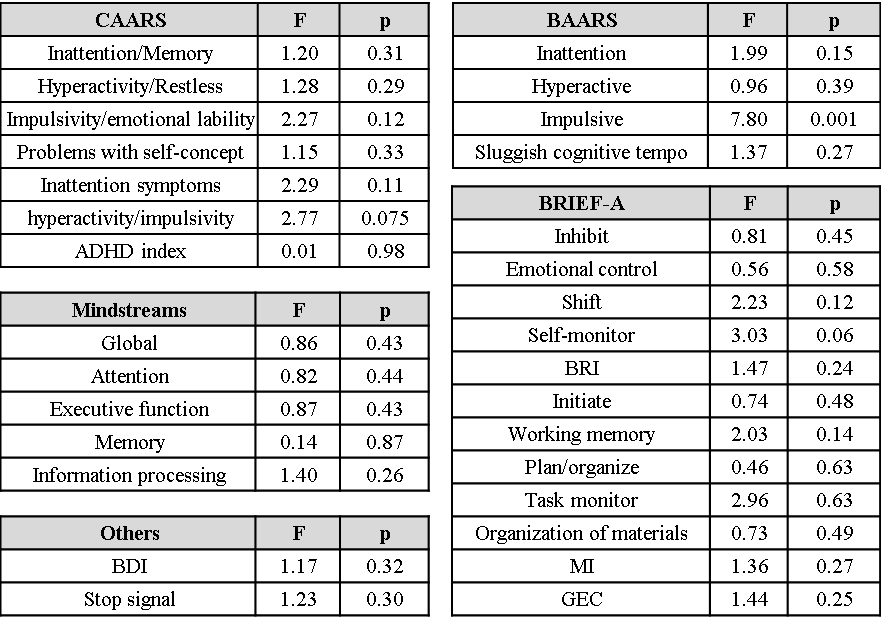


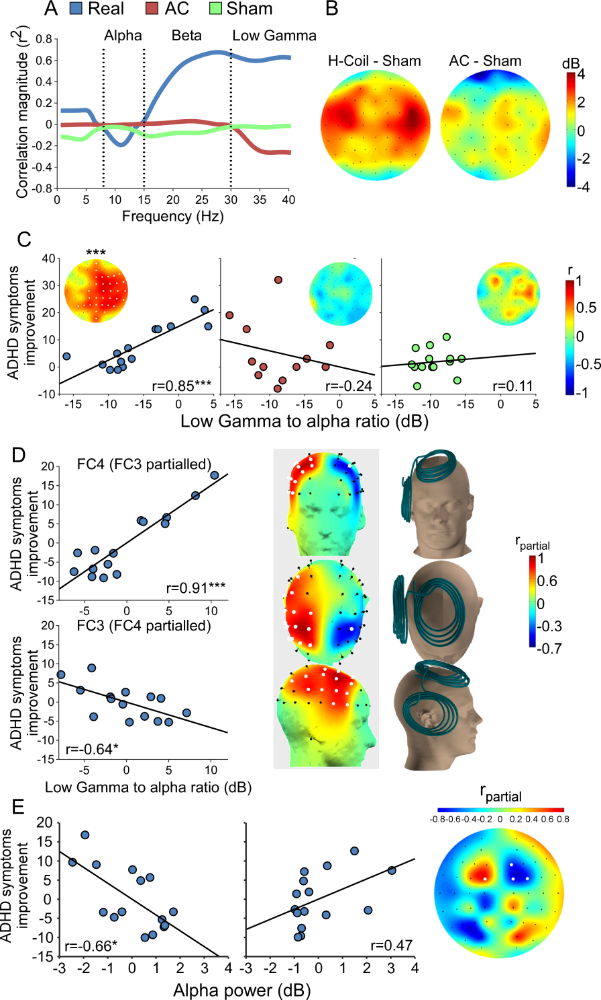


**Fig. S3**. **Stability of biomarkers under CSD (Current Source Density) transformation.** (**A**) Plot of linear correlations as a function of Group and Frequency, expressed as explained variance (r^2^; correlation sign is maintained), between activity power measured during treatment at channel FC4 (under the stimulation area) and symptoms improvement. (**B**) Topographic plots of the averaged group differences in the power of the marker (Low-gamma to Alpha power ratio), as a contrast between the active groups and the Sham group. (**C**) Topographic plots and scatter plots (at channel FC4) of the linear correlations between the marker's power and improvement of ADHD symptoms. (**D**) Scatter plots (for channels FC4 and FC3) and head plots of the marker’s inter-hemispheric balance model in the Real group. (**E**) Scatter plots (for channels FC4 and FC3) and topoplot of Alpha power inter-hemispheric balance model of resting state activity in the Real group. Significant electrodes are colored white, *p_c_<0.05, ***p_c_<0.005. AC – Active Control.


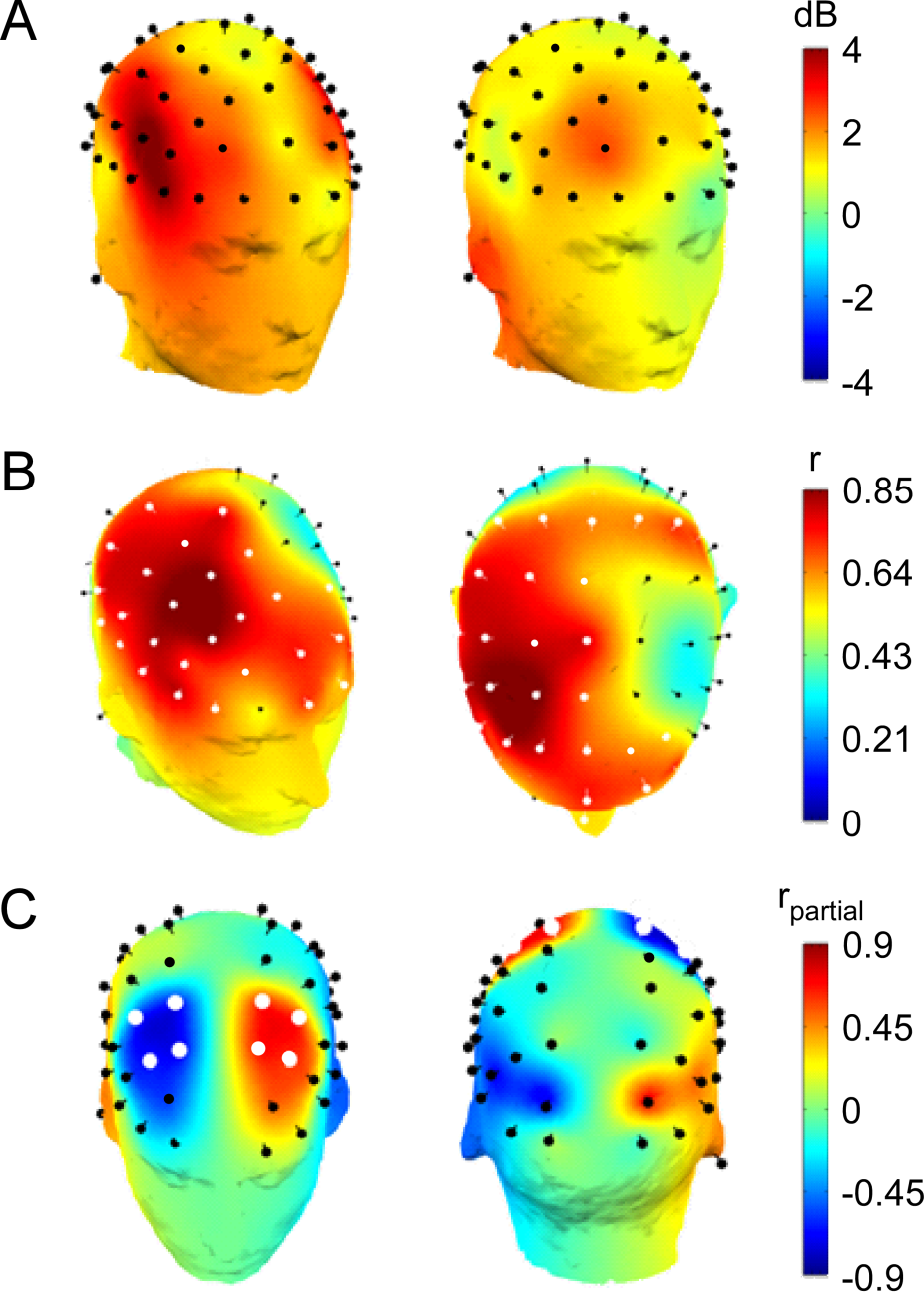


**Fig. S4. 3D head plots.** (**A**) Head plots of averaged marker’s power during treatment in the Real (left) and the AC (right) groups. (**B**) Correlations between the marker's power and improvement in ADHD symptoms in the Real group. Note that the color scale is imbalanced to emphasize the moderation of correlation magnitudes observed in the left frontal electrodes. (**C**) Inter-hemispheric balance analysis of partial correlations between Alpha power during resting state and improvement in ADHD symptoms in the Real group. Electrodes for which correlations are significant are colored white.


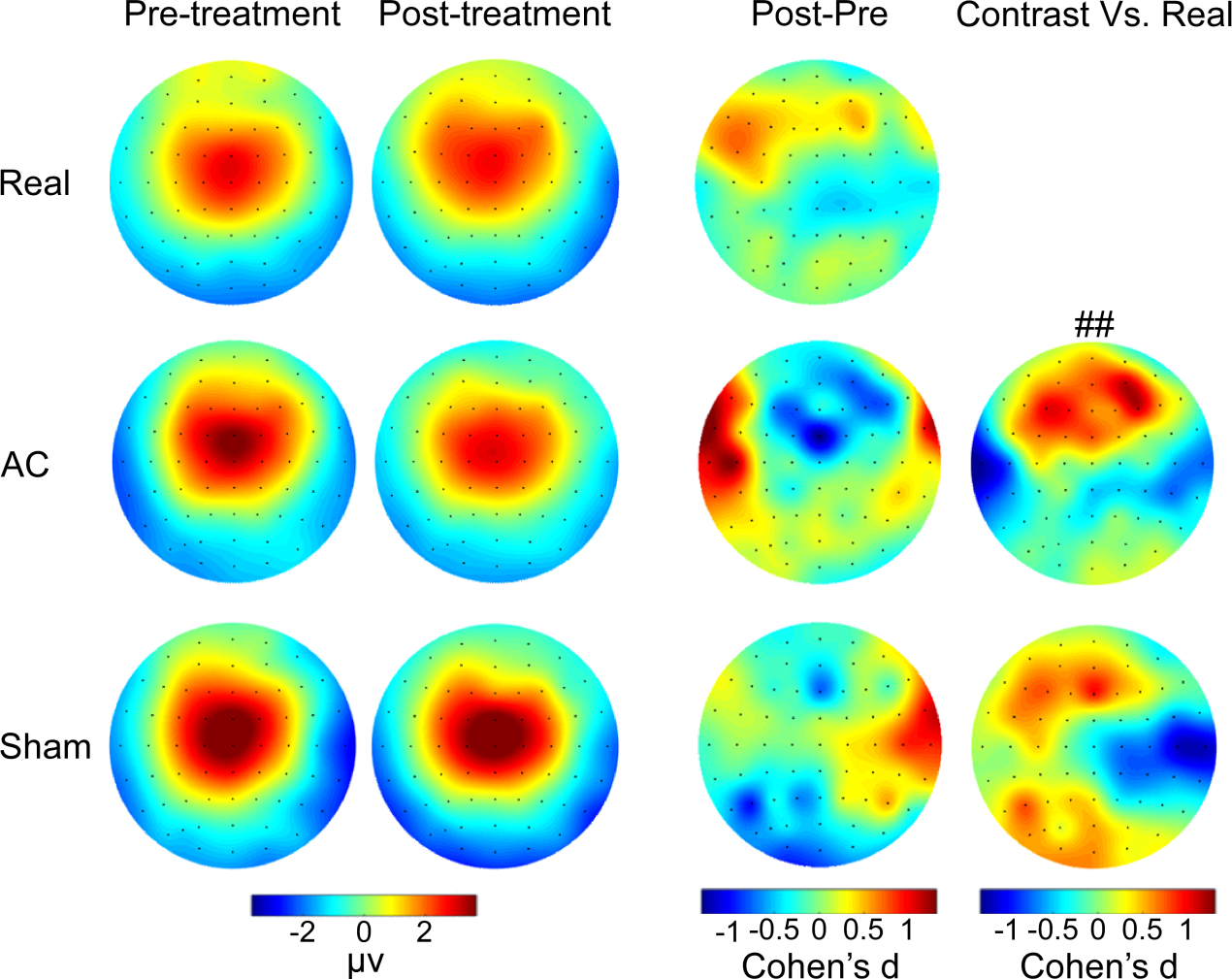


**Fig. S5**. **Long term influence of rTMS treatment on the TEP P180 component.** Topographic plots of the averaged TEPs at the P180 TOI are presented for Pre- and Post-treatment, for Post- minus Pre-treatment, and for the contrast between the control groups and the Real group (expressed in effect size; Cohen’s d). A local Time X Group interaction was found in two electrodes placed under the stimulation area (channel F4, p_uc_=0.028; channel AF4, p_uc_=0.049), but no significant cluster was identified. ^##^p<0.05 in channels F4 and AF4. AC – Active Control.


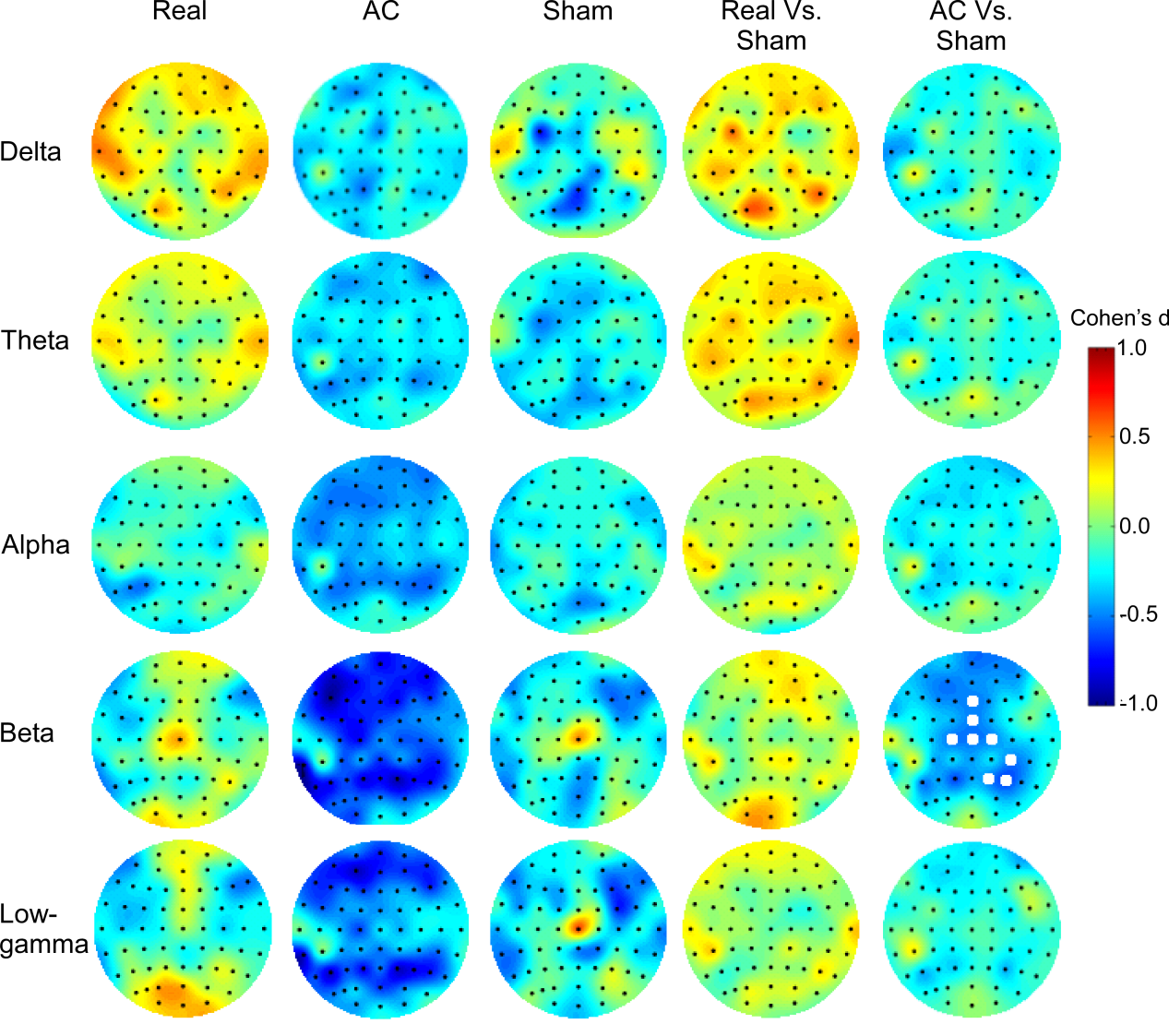


**Fig. S6. Long term influence of rTMS treatment on resting state EEG activity.** Topographic plots of differences in resting state activity power, expressed as Cohen’s d, between Post and Pre-treatment recordings in the Real, AC and Sham groups (3 left columns). Also shown pairwise contrasts between the Real group (second column from the right), AC group (most right column) and the Sham group. Significant cluster (p_c_=0.027) of power reduction in beta activity was identified in the AC-Sham contrast. This was observed in electrodes not under the stimulation area and is probably not related to the clinical effect of the rTMS as the AC group did not improve in ADHD symptoms. AC – Active Control.

**References**

Benjamini, Y., Hochberg, Y., 1995. Controlling the false discovery rate: a practical and powerful approach to multiple testing. J. R. Stat. Soc. Ser. B Methodol. 57, 289–300.

Berger, A., Alyagon, U., Hadaya, H., Atzaba-Poria, N., Auerbach, J.G., 2013. Response inhibition in preschoolers at familial risk for attention deficit hyperactivity disorder: a behavioral and electrophysiological stop-signal study. Child Dev. 84, 1616–1632. https://doi.org/10.1111/cdev.12072

Conners, C.K., Erhardt, D., Sparrow, E., 1999. CAARS: technical manual. Multi-Health Systems Incorporated (MHS).

Delorme, A., Makeig, S., 2004. EEGLAB: an open source toolbox for analysis of single-trial EEG dynamics including independent component analysis. J. Neurosci. Methods 134, 9–21. https://doi.org/10.1016/j.jneumeth.2003.10.009

Doniger, G.M., 2008. Mindstreams: guide to normative data.

Hadar, A., Hadas, I., Lazarovits, A., Alyagon, U., Eliraz, D., Zangen, A., 2017. Answering the missed call: Initial exploration of cognitive and electrophysiological changes associated with smartphone use and abuse. PLOS ONE 12, e0180094. https://doi.org/10.1371/journal.pone.0180094

Jung, T.-P., Makeig, S., Humphries, C., Lee, T.-W., McKEOWN, M.J., Iragui, V., Sejnowski, T.J., 2000. Removing electroencephalographic artifacts by blind source separation. Psychophysiology 37, 163–178.

Kähkönen, S., Komssi, S., Wilenius, J., Ilmoniemi, R.J., 2005. Prefrontal transcranial magnetic stimulation produces intensity-dependent EEG responses in humans. NeuroImage 24, 955–960. https://doi.org/10.1016/j.neuroimage.2004.09.048

Maris, E., Oostenveld, R., 2007. Nonparametric statistical testing of EEG- and MEG-data. J. Neurosci. Methods 164, 177–190. https://doi.org/10.1016/j.jneumeth.2007.03.024

Oostenveld, R., Fries, P., Maris, E., Schoffelen, J.-M., 2011. FieldTrip: open source software for advanced analysis of MEG, EEG, and invasive electrophysiological data. Intell Neurosci. 2011, 1:1–1:9. https://doi.org/10.1155/2011/156869

Premoli, I., Castellanos, N., Rivolta, D., Belardinelli, P., Bajo, R., Zipser, C., Espenhahn, S., Heidegger, T., Müller-Dahlhaus, F., Ziemann, U., 2014. TMS-EEG signatures of GABAergic neurotransmission in the human cortex. J. Neurosci. 34, 5603–5612. https://doi.org/10.1523/JNEUROSCI.5089-13.2014

Rogasch, N.C., Sullivan, C., Thomson, R.H., Rose, N.S., Bailey, N.W., Fitzgerald, P.B., Farzan, F., Hernandez-Pavon, J.C., 2017. Analysing concurrent transcranial magnetic stimulation and electroencephalographic data: A review and introduction to the open-source TESA software. NeuroImage 147, 934–951. https://doi.org/10.1016/j.neuroimage.2016.10.031

Rogasch, N.C., Thomson, R.H., Farzan, F., Fitzgibbon, B.M., Bailey, N.W., Hernandez-Pavon, J.C., Daskalakis, Z.J., Fitzgerald, P.B., 2014. Removing artefacts from TMS-EEG recordings using independent component analysis: importance for assessing prefrontal and motor cortex network properties. NeuroImage 101, 425–439. https://doi.org/10.1016/j.neuroimage.2014.07.037

STATISTICA (data analysis software system), version 13, 2016. . Dell Inc.

Wheeler, R.E., Davidson, R.J., Tomarken, A.J., 1993. Frontal brain asymmetry and emotional reactivity: a biological substrate of affective style. Psychophysiology 30, 82–89. https://doi.org/10.1111/j.1469-8986.1993.tb03207.x
